# Proton Exchange Membrane Fuel Cells (PEMFCs): Advances and Challenges

**DOI:** 10.3390/polym13183064

**Published:** 2021-09-10

**Authors:** Miriam M. Tellez-Cruz, Jorge Escorihuela, Omar Solorza-Feria, Vicente Compañ

**Affiliations:** 1 Department of Chemistry, Centro de Investigación y de Estudios Avanzados, Av. IPN 2508, Ciudad de México 07360, Mexico; mtellez@cinvestav.mx (M.M.T.-C.); osolorza@cinvestav.mx (O.S.-F.); 2 Departamento de Química Orgánica, Universitat de València, Av. Vicent Andrés Estellés s/n, Burjassot, 46100 Valencia, Spain; 3 Departamento de Termodinámica Aplicada (ETSII), Universitat Politècnica de València, Camino de Vera s/n, 46022 Valencia, Spain

**Keywords:** proton exchange membrane, fuel cell, membrane–electrode assembly, organic polymers, proton conductivity

## Abstract

The study of the electrochemical catalyst conversion of renewable electricity and carbon oxides into chemical fuels attracts a great deal of attention by different researchers. The main role of this process is in mitigating the worldwide energy crisis through a closed technological carbon cycle, where chemical fuels, such as hydrogen, are stored and reconverted to electricity via electrochemical reaction processes in fuel cells. The scientific community focuses its efforts on the development of high-performance polymeric membranes together with nanomaterials with high catalytic activity and stability in order to reduce the platinum group metal applied as a cathode to build stacks of proton exchange membrane fuel cells (PEMFCs) to work at low and moderate temperatures. The design of new conductive membranes and nanoparticles (NPs) whose morphology directly affects their catalytic properties is of utmost importance. Nanoparticle morphologies, like cubes, octahedrons, icosahedrons, bipyramids, plates, and polyhedrons, among others, are widely studied for catalysis applications. The recent progress around the high catalytic activity has focused on the stabilizing agents and their potential impact on nanomaterial synthesis to induce changes in the morphology of NPs.

## 1. Introduction

**Figure 1:**
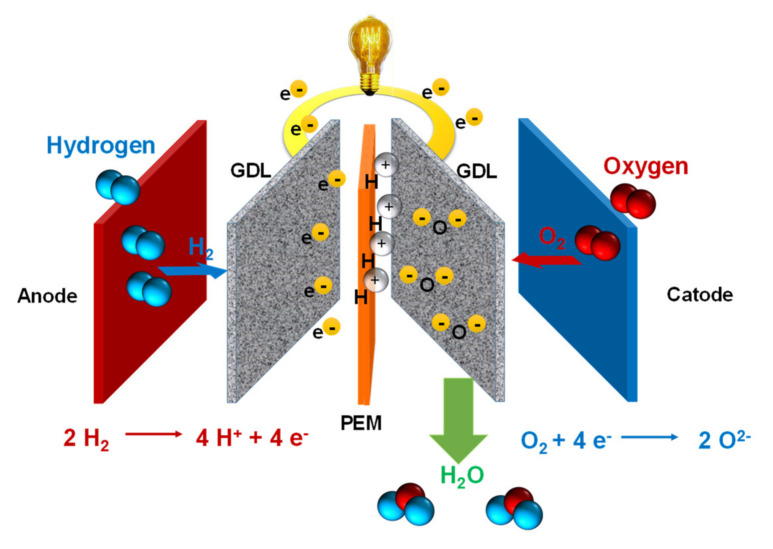
Schematic diagram showing the components of a single PEMFC: anode, cathode, gas diffusion layer (GDL), membrane, anode, and cathode reactions together and products.

The study of proton exchange membrane fuel cells (PEMFCs) has received intense attention due to their wide and diverse applications in chemical sensors, electrochemical devices, batteries, supercapacitors, and power generation, which has led to the design of membrane-electrode assemblies (MEAs) that operate in different fuel cell types [1,2,3]. Fuel cells based on proton exchange membranes (PEMs) are among the most promising electrochemical-generating devices due to their high efficiency, high power density, low emissions, and energy supply [4,5]. Even when compared to devices such as Redox flow batteries (RFBs), they share practically the same configuration. Although both types of devices allow the chemical energy contained in energy vectors obtained from renewable sources to be converted into electricity, PEMFCs have advantages over RFBs, such as the absence of liquid components (which makes their use in mobile devices practical), there are no toxic components outside the cell (compared to vanadium RFB), there are no precipitation reactions that limit their energy density, they do not have electrolytes with high ohmic resistance (non-aqueous electrolytes) that can present problems of evaporation and instability, nor they present problems of dendritic growth of metals that represent safety problems, in addition to presenting a much greater long-term operating stability [6]. Figure 1 shows a schematic diagram showing the components of a single PEMFC. These alternative energy sources provide the possibility of receiving energy from hydrogen and synthetic or bio-synthetic fuel and can operate with greater efficiency and environmental sustainability compared to thermal motors [7,8]. Fuel cells are electrochemical devices used for various technological applications, such as in vehicles, mobile phones, portable electronics, and power generators [9,10,11].

In a typical PEMFC, the polymer electrolyte membrane is responsible for the proton conductivity that allows the transport of protons from the anode to the cathode, constituting the essential component of the electrochemical device [12]. Among the diverse types of fuel cells, membranes based on perfluorosulfonic acid polymers, such as Nafion^®^, are successfully used due to their high conductivity and good chemical and mechanical properties; these are used at temperatures below 90 °C and conditions of high relative humidity [13,14]. Nafion^®^ was developed by DuPont in the late 1960s and is still the state-of-the-art low-temperature PEM. The main drawbacks of Nafion membranes for operation as low-temperature PEMFCs (LT-PEMFCs) are mainly their expensive manufacturing processes and the strong decrease in proton conductivity at temperatures above 90 °C, when low hydration conditions are attained as a consequence of the loss of the ion-exchange functional groups, which takes place beginning at 130 °C. These practical limitations have promoted the emergence of intermediate-temperature PEMFCs (IT-PEMFCs) and high-temperature PEMFCs (HT-PEMFCs), which operate, respectively, between 100–150 °C and 120–200 °C in the absence of water and are the focus in this review [15,16,17,18,19].

It is worth mentioning the decrease in catalytic activity due to electrode poisoning by CO and CO_2_ contamination when working at moderate and high temperatures. The electrode kinetics are faster and have simpler thermal and water handling, low dependency on cooling systems, high amounts of reusable heat energy, as well as a lower cost of the membrane-electrode assemblies (MEAs), in comparison with LT-PEMFCs based on Nafion polymers [15,16,17,18,19]. The high CO tolerance of the anode catalysts makes it possible for a FC to use hydrogen directly from a simple methanol reformer, so that the selective oxidant and/or the CO separator of the membrane can be simplified or removed from the processing system. Consequently, the size and volume of a fuel cell is reduced to improve its performance, responsiveness, and reliability, which ultimately allows reducing system maintenance and operation costs [20]. A lot of work has been done in order to optimize the performance of FCs in the development of IT-PEMs and HT-PEMs, particularly based on sulfonated polyether ether ketone (SPEEK) and polybenzimidazoles (PBIs), among others, which have emerged as promising candidates to operate at moderate and high temperatures and reach high conductivity under anhydrous conditions [21,22,23,24,25].

With the increasing worldwide demands in energy, consumption and increasing environmental concerns results play a vital role in the development of clean and renewable energy sources as a substitute for the traditional consumption of fossil fuels. Energy generation and consumption have largely relied on burning fossil fuels, thus increasing the levels of CO_2_ in the atmosphere and causing serious environmental problems, including global warming and climate changes [26,27,28,29,30,31]. The electrochemical catalytic conversion of renewable electricity and carbon oxides into chemicals and fuels has attracted a great deal of attention as it may contribute to mitigating the worldwide energy crisis through the use of a closed technological carbon cycle, where chemical fuels such as hydrogen, can be stored and reconverted into electricity via electrochemical reaction processes in fuel cells [32,33,34]. There has been increasing interest in the development of PEMFCs because they are considered as the most promising clean future energy source for applications in industries, portable equipment, and as an alternative powertrain for transportation [35,36].

The electrocatalyst is a key component for securing a hydrogen-based clean and sustainable energy cycle. The definitive test for a new electrode material is to determine the reactivity as stated by the Sabatier principle and the catalytic activity of the solid surfaces combined with density functional theory (DFT) to predict how it will perform in a wealth of full-scale applications of fuel cells. There has been extensive effort in developing low-cost, efficient electrocatalysts for electrolyzers and fuel cells through inexpensive, highly active, abundant, environmentally friendly, and stable electrocatalysts for mass device production. New electrocatalytic nanomaterials, such as metals, metal oxides, and non-metals have been widely synthesized in different media, focusing on understanding how to control the geometric shape, sizes, composition, architecture, and micro-/nanostructures. They provide an effective strategy to control their reactivity and catalytic properties for their applications as electrode materials in electrochemical reactions, in electrical energy storage devices, and in fuel cells [37,38,39]. Doping or coupling another metal to form an alloy could reconstruct the catalyst surface state, which could change the binding energy of intermediates on the surface during the reaction [40,41].

The goal of this review was to give an overview of the experimental synthesis procedure of the components of the H_2_/O_2_ PEMFC and an evaluation of the material’s performance as reported recently in the literature, which could contribute to progress in this field. One of the challenges of the operation of low-temperature fuel cells is overcoming the sluggish ORR rate, which is at least four orders of magnitude slower than the HOR. This electrochemical property imposes performance limits on the global process and requires the use of catalytic materials with highly specific and mass catalytic activities, as well as stability, durability, and selectivity at the cathodic side to accelerate the oxygen reduction in energy conversion devices. The basic electrochemical reactions occurring simultaneously on both sides at the electrodes of the membrane-electrode assembly, MEA, of the PEMFCs are [42]:

*Anode*  2H_2_ + 4nH_2_O → 4H^+^·nH_2_O + 4e^−^ (1)

*Cathode*  O_2_ + 4H^+^·nH_2_O + 4e^−^ → (n + 2) H_2_O (2)

*Overall*  2 H_2_ + O_2_ → 2 H_2_O (3)

**Figure 2:**
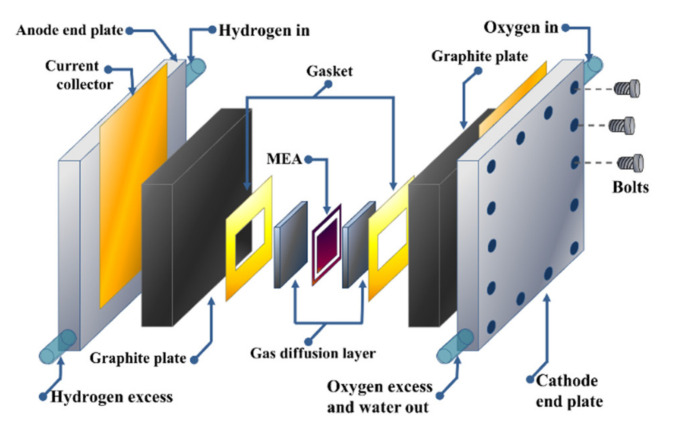
Schematic diagram showing the components of a single PEMFC: bipolar plates, gaskets, gas diffusion layers, and the MEA; this arrangement is repeated in a fuel cell stack. Reproduced with permission from Elsevier (reference [43]).

Figure 2 shows the different components of a single PEMFC. The core of a PEMFC is the MEA, formed by two catalytic materials that act as anodic and cathodic electrodes, separated by a PEM. The energy conversion is performed by means of two half-reactions: the hydrogen oxidation reaction (HOR) and the oxygen reduction reaction (ORR).

Commonly, platinum nanocatalysts and their alloys present high catalytic activities, stability, durability, and selectivity for the cathodic reaction in these energy conversion devices. The proton produced as hydrogen oxidized at the anodic side is transported through the membrane toward the cathode in the form of species H+·nH_2_O, reacting in a multi-electron charge transfer process to water formation.

**Figure 3:**
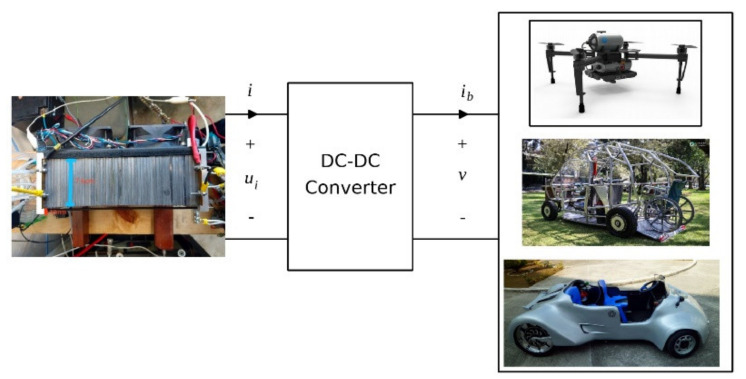
PEMFC system, DC–DC converter with diverse applications.

The simple structure of hydrogen-oxygen fuel cells has allowed for the design, manufacture, and creation of a rapid development of low- and high-power devices. These electrochemical devices provide uninterrupted electrical energy for most applications, including for portable, mining, and farming needs, as well as emergency power generators. PEMFCs also possess other notable advantages: low working temperatures, high efficiency, minimal maintenance, long service lifetime, and compactness. Additionally, the lack of moving parts in a PEMFC allows for noiseless operation. Individual single fuel cells are each capable of producing an electric potential of less than 1 V, and when much higher voltage and electrical power is required, the appropriate number of individual cells are connected. By connecting individual cells in series, an arrangement of electronically conducting bipolar plates with membrane–electrode assemblies in between is formed, where one side of such an electrode is working as the anode and the other side is working as the cathode of the neighboring cell [44]. A complete fuel cell system is shown in Figure 3, integrating a hydrogen fuel cell, a DC converter, and electric loads corresponding to various applications.

The DC-DC converters are of paramount importance to interface the fuel cell with different loading applications. The DC–DC boost converter forms an integral part of fuel cell power modulator conditioning unit, essential for compact design of the power control unit. The DC-DC boost converter and the controller are basic and imperative parts of fuel cell power modulator conditioning unit, required for compact design of the power control unit with output support for supplying the load. The boost converter offers higher efficiency and less component counts compared to other DC/DC converter topologies. Many DC-DC three-port converters have been proposed and reported [45,46,47,48], including DC/DC boost converters for electric vehicle fuel cell fuel cell applications.

PEMFCs have reached a level of development on different scalable volume from which it is possible to indicate that have promise in any market in which electricity is produced, and they are classified as portable and non-stationary devices—in both cases they operate with high efficiency and low environmental impact [49]. Globally, fuel cells have seen marked size increases in terms to their power output, with existing systems capable of operating at less than 5 W and up to several kilowatts. Standard output classifications are <2, 10–50, and 100–250 W systems [50]. Portable hydrogen fuel cell technology has drawn significant attention due to its simplicity, mass implementation feasibility, fast start-stop cycles, and a wide spectrum of possible power applications [49,51]. Fuel cell cost has, up until now, been one barrier facing the commercialization of fuel cell technology in different applications. Also, fueling fuel cells is another problem, considering the production, transportation, distribution, and storage of reactant is still technically challenging. Other limitations that need to be taken into account include the durability and reliability of the fuel cell system. Although these kinds of electricity generators have drawn growing interest, they are also considered as one as the most promising devices for standalone/grid-connected distributed generation with capability for low- and high-power applications on diverse devices and transformation potential in function of the required power. For this purpose, the fuel cell system will have a determined number of single cells associated in series (a stack of PEMFCs), along with supporting components to produce electricity of the desired voltage and current to produce the optimum power required in each case.

## 2. Development of Proton Exchange Membranes (PEM)

In the past few decades, the number of studies devoted to the development of novel conducting membranes for PEMFC applications has experienced exponential growth [52]. The need to reduce the CO_2_ concentration in the atmosphere in combination with the interest of developing new environmentally friendly alternatives to conventional fossil fuel has motivated researchers to synthesize polymers with high proton conductivity that are chemically and thermally stable and capable of operating in a wide range of different temperatures while maintaining these properties during long-term operation cycles. Currently, among the plethora of PEMFCs synthesized for this purpose, three main families need to be highlighted [53]. The first one is that based on poly(perfluorosulfonic acid)-type polymers. Among these fluorine-containing polymers, Nafion is the most used and studied polymer and constitutes the benchmark in the fuel cell industry [54]. Its use has been extended to a wide variety of applications, including microbial fuel cells [55,56] in chlor-alkali processing technologies [57,58], among others, and commercial Nafion membranes with different thicknesses can be purchased from several companies. The thickness of Nafion membranes has a strong influence on the physical and chemical properties of this fluorine-containing polymer, as demonstrated in the performance of the iron–chromium redox flow battery (ICRFB), where thinner membranes were more appropriate for the ICRFB cycling operation [59]

This sulfonated tetrafluoroethylene-based polymer has demonstrated elevated chemical and mechanical properties and excellent conductivity at temperatures below 90 °C and in high relative humidity conditions. Under these operating conditions, the proton conductivity of a Nafion membrane can reach values up to 0.1–0.2 S/cm depending on temperature and relative humidity. However, this value decreases dramatically at temperatures higher than 90 °C, mainly because of the loss of water molecules from the Nafion [60]. Nevertheless, there are benefits of operating at higher temperatures, such as reduction in catalyst poisoning, faster electrode kinetics, and ease associated with water and thermal management [61]. Consequently, most efforts have been diverged to the development of new types of HT membranes that can be used under anhydrous conditions, such as intermediate-temperature PEMFCs (IT-PEMFCs) and high-temperature PEMFCs (HT-PEMFCs), which operate in the intervals of 90–120 °C and 140 °C up to 200 °C, respectively. This limitation restricts the use of Nafion membranes that are only suitable for low-temperature PEMFCs (LT-PEMFCs), which operate around 50–90 °C.

The second main group of conducting materials is based on sulfonated aromatic polymers. The sulfonated polymers which have been most widely studied for fuel cell applications are sulfonated poly(arylene ether ketone) (SPAEK) and sulfonated poly(ether ether ketone) (SPEEK). Poly(ether ether ketone) (PEEK) is a semicrystalline polymer with a melting point of 343 °C and a glass transition temperature of 143 °C, based on a linear polymeric backbone in which 1,4-disubstituted phenyl groups are linked by ether and carbonyl groups. This nonfluorinated polymer possesses high thermal stability, chemical resistance, and its proton conductivity is enhanced significantly by sulfonation of the aromatic position [62], which should enable operation at elevated temperatures in which electrochemical reaction rates speed up. This chemical modification increases the acidity and hydrophilicity, therefore facilitating the proton transport as it favors the interaction with water molecules. It is known that Tg values of SPEEK depend on sulfonation degree, i.e., ion-exchange capacity (IEC), and glass transition temperatures above 170 °C are usually found [62,63]. Therefore, SPEEK membranes have sufficient stability to operate at intermediate temperatures (120–140 °C) in PEMFC.

In the last decade, our group of research has focused on the characterization of SPEEK membranes with two different sulfonation degrees (1.75 and 2.05 meq/g ion-exchange capacity). Their utilization is in microbial fuel cells (MFCs) operating at ambient temperatures, although they could also be used at any temperature below 150 °C, given that at these conditions both pristine SPEEK membranes become water soluble. We also have studied the effect of blending SPEEK with two polymers, polyvinyl alcohol (PVA) and polyvinyl butyral (PVB), with the aim to promote crosslinking and enhance the mechanical stability of the pristine membranes. Water uptake is correlated with the ion-exchange capacity (IEC) and is the main parameter to determine the properties of mechanical stability and proton conductivity. Interestingly, for the same membrane, IEC was found to be dependent on the membrane history, which we explained is due to the peculiar morphology exhibited by the SPEEK materials, that is, narrower and shorter with less interconnected ionic channels for proton conduction than Nafion^®^ [64].

Two main approaches have been described for the SPEEK preparation: (i) via direct polymerization from the sulfonated monomer [65] or (ii) by sulfonation reaction of the polymeric backbone [66]. The sulfonation of PEEK to develops SPEEK, which maintains the good mechanical, thermal, and chemical stability of PEEK and, interestingly, displays high proton conductivity values (0.10–0.18 S/cm), however, SPEEK conductivity decreases at temperatures over 120 °C, and therefore, the optimal operating temperature is generally limited to 80–120 °C. Another limiting factor that has reduced its use in comparison with perfluorinated-based membranes is the membrane lifetime, as its durability is inferior to that of Nafion under similar fuel cell operating conditions. In this context, SPEEK appears to be unsuitable for PEM because of limited membrane lifetime, but similar polymers, such as sulfonated aromatic polymers, e.g., polyphenylene, have shown improved stability under working conditions [67,68,69].

**Figure 4:**
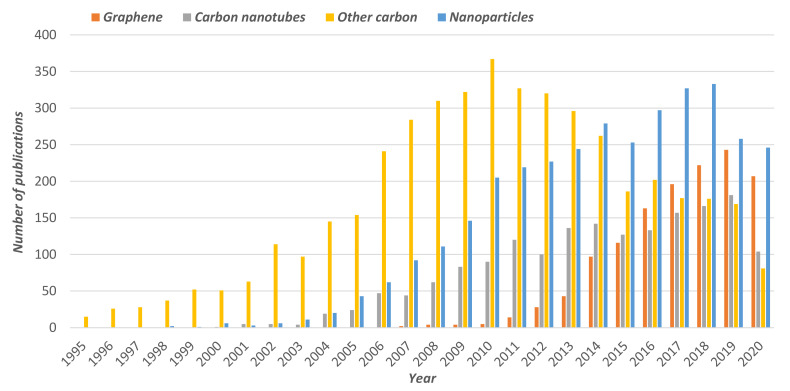

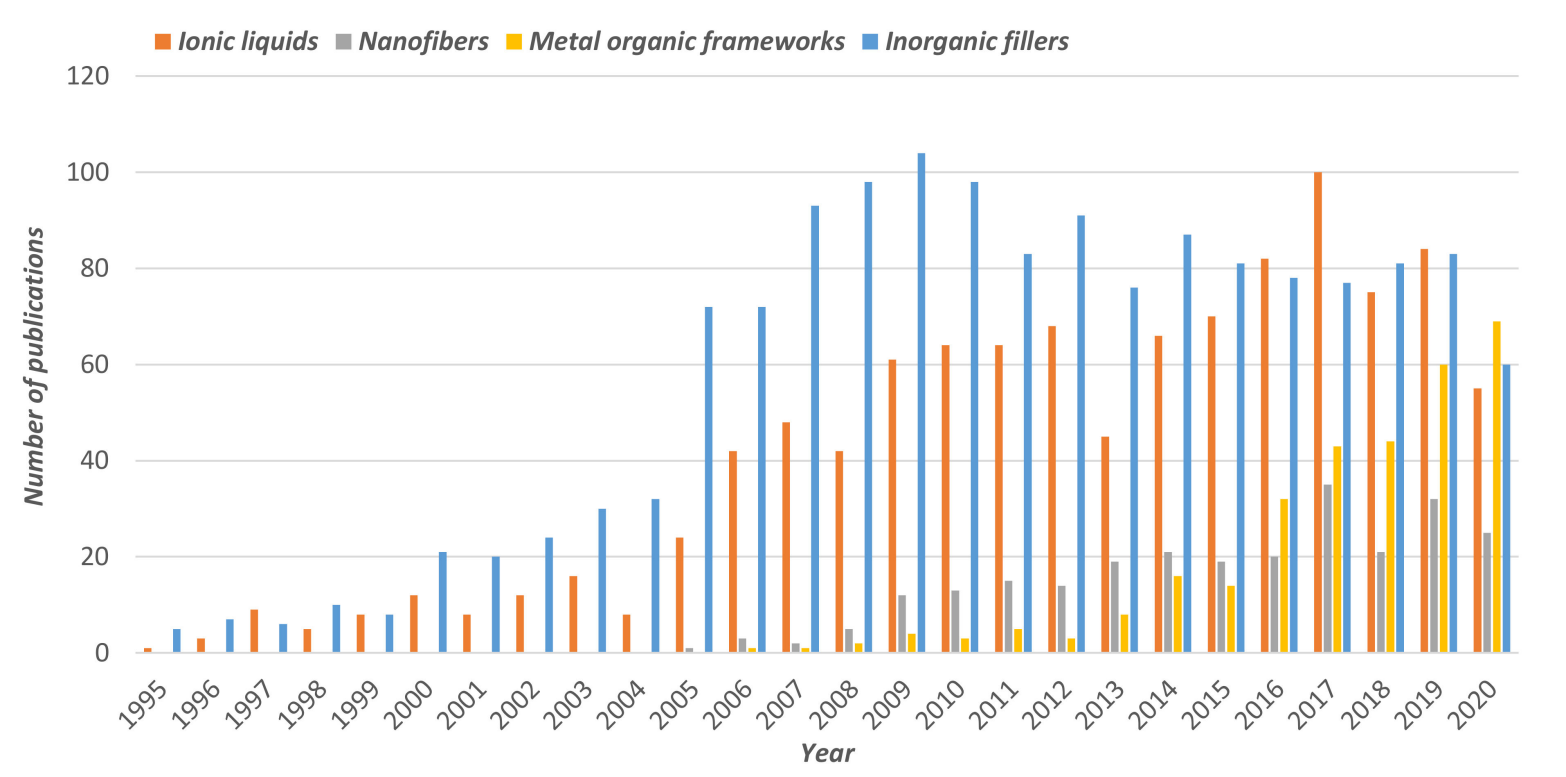
Number of publications in the period of 1995–2020 (until September) indexed in the Web of Science: keywords: proton exchange membrane AND filler (graphene, carbon nanotube, other carbon fillers, nanoparticles, nanofibers, metal organic frameworks, and inorganic fillers). Source: www.webofknowledge.com (accessed on 1 February 2021).

The third and last class of polymers that will be discussed here are those based on heterocyclic systems, which include the derivates of polybenzimidazole (PBI) [70]. PBI is a heterocyclic aromatic polymer that can be easily prepared via a polycondensation reaction of 3,3′-diaminobenzidine (DAB) with isophthalic acid (IPA). Interestingly, a few new types of branched PBI-based polymers have been synthesized in recent years, showing good performances as PEMFCs [71,72,73]. In general, PBI-based polymers possess a high resistance to acidic and basic inorganic reagents, with elevated glass transition temperatures (425–436 °C) and excellent thermal and mechanical stability [74]. Given their elevated thermal stability, PBI polymers and their derivatives have emerged as potential candidates to be used as HT-PEMFCs. Despite its excellent thermal and mechanical stability, the conductivity of pristine PBI is low and these membranes require doping with inorganic acids, such sulfuric acid, nitric acid, chlorohydric acid, and, by far the most widely used, phosphoric acid. Under this acidic doping, conductivities around 0.1 S/cm can be easily achieved, even under dry conditions. The main drawback of using mineral inorganic acids is the acid leaching, which can cause an important degradation of the membrane when scaled at the electrode assembly (MEA) in fuel cell systems. Although alternative acids have also been studied, such as phytic and phosphotungstic acids, the conductivity values are very far from those obtained with phosphoric acid [23,75,76,77,78,79,80,81]. All the above-mentioned type of membranes have been used in combination with different type of fillers with the intention of enhancing the proton conductivity of the membranes. This quest has led to the preparation of mixed-matrix membranes (MMMs), which generally contain a bulk continuous polymer phase and a dispersed inorganic or organic dispersed phase and combine the benefits of both materials [82]. As displayed in Figure 4, different fillers have efficiently been used for this purpose, as reflected in the number of publications within the past 25 years. A closer look at this figure reveals that the use of fillers such as graphene, carbon nanotubes, metal organic frameworks, and ionic liquids is still growing up and novel MMMs containing these fillers are under development.

In the next sections, the most representative membranes using some of the above-mentioned fillers with the three main families of polymeric materials, namely Nafion, SPEEK, and PBI, will be briefly discussed.

### 2.1. Graphene and Carbon Nanotubes

**Figure 5:**
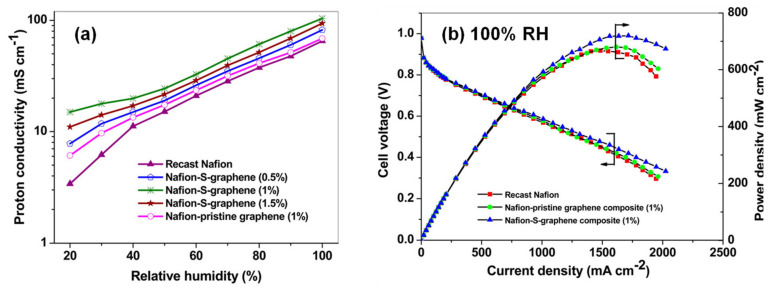
(**a**) Proton conductivity of recast Nafion, Nafion–graphene, and Nafion-S-graphene (sulfonated graphene) composite membranes as a function of relative humidity. (**b**) Performance of H_2_/O_2_ PEFC with recast Nafion, Nafion-graphene, and Nafion-S-graphene composite membranes at 100% RH at 70 °C under atmospheric pressure. Reprinted with permission from reference [94]. Copyright 2016 American Chemical Society.

Among the different carbon-derived materials, graphene and carbon nanotubes have been positioned as the most common fillers used to enhance proton conductivity in polymeric membranes. Graphene is a two-dimensional sheet of sp^2^-hybridized carbon that has been widely used in many technological areas, such as nanoelectronics [83], electrochemistry [84], catalysis [85,86], sensors [87], adsorption [88], and energy storage [89]. This material possesses excellent thermomechanical stability, high electrical conductivity, and a large number of active sites, which allow further chemical modifications and the introduction of a functional site [90]. Composite Nafion membranes with graphene layers have been reported to improve conductivity values up to 0.14 S/cm under low-humidity conditions [91]. Graphene oxide (GO) can be easily obtained from the oxidation of graphite and is based on 2D carbon sheets with oxygen-containing functionalities [92]. The use of GO has also been explored and MEAs fabricated with Nafion containing GO displayed excellent cell performances, reaching values up to 1.27 A/cm^2^ at RH 100% compared to 0.435 A/cm^2^ for the pristine Nafion membrane [93]. Some chemical modifications of graphene, such as sulfonation of graphene, increased the proton conductivity of Nafion composite membranes by about five times over that of a pristine Nafion membrane under low-humidity conditions and a peak power density of 300 mW/cm^2^ at a load current density of 760 m A/cm^2^ at 70 °C and 20% RH (Figure 5) [94,95].

Graphene and GO have also been used in combination with SPEEK membranes and several membranes with enhanced stability and proton conductivity have been reported. In this regard, conductivity values close to that of Nafion-based membranes can be achieved. Thus, sulfonated graphene (SG) was cross-linked with SPEEK and the resulting membrane exhibited a conductivity up to 0.085 S/cm [96,97]. Sulfonic-acid-functionalized GO has also yielded membranes with enhanced conductivity [98,99].

**Figure 6:**
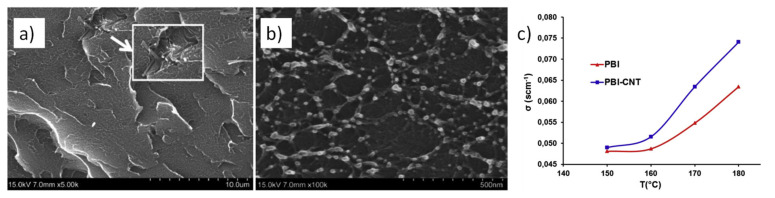
(**a**) FE-SEM micrograph of PBI-CNT cross-section. (**b**) Magnification of FE-SEM micrograph of PBI-CNT cross-section. (**c**) Temperature dependence of proton conductivity of PBI and PBI-CNT membranes. Reprinted with permission from reference [99]. Copyright 2001 Elsevier.

On one hand, the combination of PBI and carbon-based materials, such as graphene, has afforded the fabrication of composite membranes with enhanced proton conductivities with values of 0.17 S/cm at 165 °C when a 2 wt.% GO loading was used and displaying peak power densities of 0.38 W/cm^2^ [100]. Furthermore, GO has been modified with a wide variety of groups in order to improve the membrane properties. In this regard, sulfonated GO afforded proton conductivity around 0.07 S/cm at 160 °C under dry conditions [101,102]. On the other hand, the proton conductivity of PBI membranes has been enhanced using CNTs [103] and MWCNTs [104,105], reaching values up to was 0.08 S/cm (Figure 6).

### 2.2. Metal. Organic Frameworks

**Figure 7:**
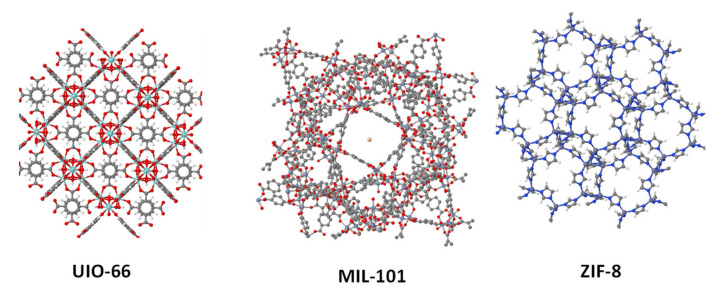
Structures of the MOFs most widely used as fillers in PEMS.

MOFs are crystalline solids built by the connection of metallic atoms and organic linkers yielding porous structures with cages, channels, or cavities, which have been considered as highly promising porous materials for a large variety of applications [106,107]. Up until now, more than 90,000 different MOF structures have been identified and their use in energy applications has blossomed in the last few decades [108,109,110]. MOFs have been used as efficient fillers with a wide variety of polymeric materials, including Nafion, SPEEK, and PBI, but also vinyl-type polymers, such as poly(vinylalcohol) (PVA) [111,112,113] and poly(vinylidene fluoride) (PVDF), [114,115] chitosan [116], and polyetherimide (PEI) [117]. A series of MOF structures is presented in Figure 7, displaying the most common MOFs used as fillers in PEMS for fuel cell applications.

Among the wide diversity among all MOF subclasses, two different MOF units have been more widely studied in the preparation of MMMs for energy applications. In first place, Zr-based MOFs have attracted considerable attention in the last decade, particularly, UiO-66, which is a Zr-based MOF constituted of hexanuclear Zr clusters linked by terephthalic acid (bdc), with triangular pores of ≈6 Å and with moderate hydrophilicity [118]. Interestingly, the size of the particle can be easily tuned by using different reaction conditions in the material synthesis, which expands its versatility to be used as a filler in composite membranes. Secondly, there are the MIL-101 derivatives, which are highly porous chromium terephthalate MOFs, whose structure was first reported by Ferey et al. in 2005 [119]. This MOF can be sulfonated without modifying the crystal structure, increasing its proton conductivity to values close to 0.01 S/cm at temperatures up 150 °C and dry conditions.

In the case of Nafion-based polymers, phytic acid was impregnated into MIL-101 cavities and used as a filler in Nafion membranes, creating MMMs with conductivities of 0.23 S/cm at 100% RH and 100 °C. Chemical modification of MIL-101 can also be performed and sulfonated MIL-101 has been used in the fabrication of Nafion composite membranes, reaching conductivity values up to 0.2 S/cm at temperatures over 100 °C, without adding any acid to the membrane [120]. This value is among the highest ever reported for PEMs and shows the potential application of this MOF for fuel cell applications. UiO-66 has also been sulfonated and used in the preparation of composite Nafion membranes with conductivities up to 0.12 S/cm at 90 °C and 95% RH [121,122].

**Figure 8:**
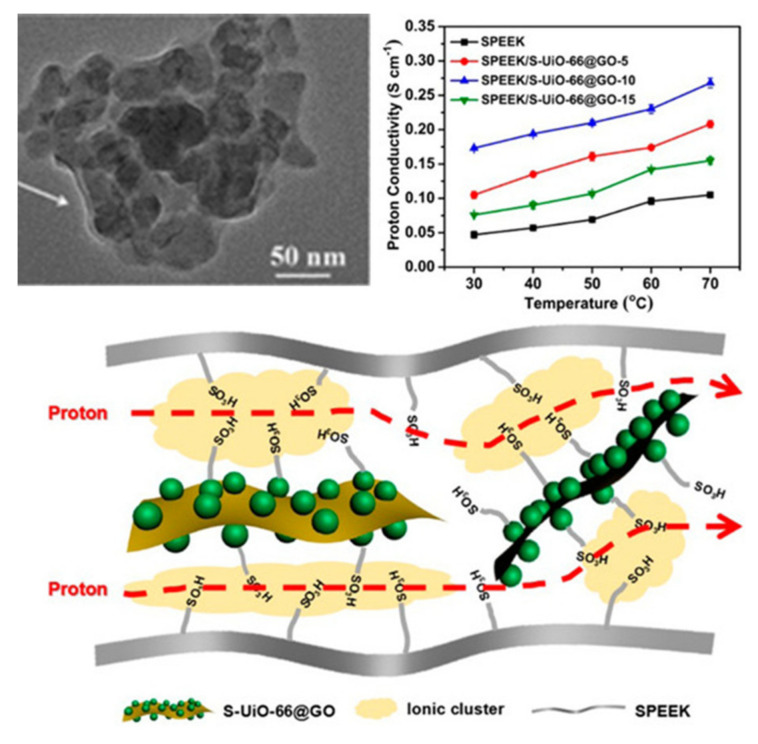
Top left: TEM images of SPEEK hybrid nanosheets with GO and UiO-66. Top right: Temperature-dependent proton conductivities under 95% RH. Bottom: Schematic illustration of the enhanced transport properties of the SPEEK/S-UiO-66@GO composite membranes. Reprinted with permission from reference [125]. Copyright 2017 American Chemical Society.

Although poly (arylene ether)-based membranes display lower proton conductivities than Nafion membranes, the incorporation of MOFs to the SPEEK matrix can have beneficial effects, increasing its proton conductivity up to 0.3 S/cm when using sulfonated MIL-101 as an acidic filler [123]. This chromium-based MOF has also been used in combination with phosphotungstic acid, reaching a maximum proton conductivity of 0.27 S/cm at 65 °C and 100% RH [124]. Zr-based MOFs have also been studied in SPEEK membranes for the preparation of MMMs containing a uniform dispersion of sulfonated UiO-66 onto GO nanosheets (Figure 8). These composite membranes showed a significant increase in their proton conductivity over the recast SPEEK, reaching values up to 0.27 S/cm at 70 °C and 95% RH [125]. Ionic liquids can also be encapsulated into the UiO-66 cavities, creating SPEEK composite membranes with conductivities up to 0.14 S/cm at 80 °C and 60% RH [126]. Although other different MOFs have been used as filler in SPEEK membranes, the conductivities were far from those achieved with the widely used MIL-101 and UiO-66 [127,128].

The number of studies based on PBI membranes with MOFs as fillers are scarce, but have shown a potential application in fuel cell technology. Zeolite imidazolate frameworks (ZIFs) have been successfully implemented as fillers in the fabrication of PBI composite membranes and compared with the single ZIF doping (ZIF-8 or ZIF-67), a combination of both materials (ZIF-mix) was shown to improve the proton conductivity of the pristine PBI membrane, which was attributed to the additional proton carriers provided from ZIFs [129]. In a recent example, a synthetically modified UiO-66-NH_2_ MOF containing acidic −SO_3_H groups was used as a filler in PBI membranes reaching proton conductivities up to 0.30 S/cm at 160 °C under anhydrous conditions [130]. ZIFS have also been used as fillers for SPEEK membranes with similar enhancing results [131]. In a recent work by Wang and coworkers, MOF UiO-66 was introduced in a crosslinked PBI membrane, reaching high proton conductivity (up to 0.100·S cm^−1^) and achieving a maximum high peak power density of 607 mW·cm^−2^ at 160 °C [132]

### 2.3. Ionic Liquids

Ionic liquids (ILs) are ionic organic compounds with generally low melting points. Ionic liquids are used as fillers with the intention to increase proton conductivity of polymeric membranes and also to improve thermal, chemical, and mechanical stability of the membranes. Ionic liquids have found tremendous applications in a wide variety of fields of chemistry, such as organic chemistry [133], which includes their use as green solvents in organic synthesis [134,135]; catalysis [136,137]; supramolecular chemistry [138,139]; pharmaceutical chemistry [140]; and as transport agents [141]. They have also been used in analytical chemistry in engineering applications, sensing, separation, extraction, and drug sensing [142,143,144,145,146,147,148,149], and also in materials science and electrochemistry [150], among other fields. ILs have several favorable properties, including their temperature stability, rather high ionic conductivity, and reduced environmental impact. Since its discovery, ILs have been considered as promising compounds for the preparation of composite membranes for energy applications [151]. Among the wide variety of ILs, those containing N-heterocyclic cations, such as imidazolium salt, have been widely used as fillers in composite polymeric membranes.

Recently, the use of ionic liquids containing heterocycles such as pyrazole, imidazole, triazole, or benzimidazole have been considered as promising candidates to enhance proton conductivity of Nafion membranes under low-humidity conditions. Doyle and coworkers used 1-butyl, 3-methyl imidazolium trifluoromethanesulfonate as a filler in Nafion membranes reaching ionic conductivities of 0.1 S/cm at 180 °C [152]. The good interaction of the polymeric matrix with the IL was crucial in enhancing the conductivity, as shown by Schäafer et al. by confocal Raman spectroscopy [153]. Schäafer and coworkers also used 1-butyl-3-methylimidazolium, but in combination with doping with phosphoric acid, which afforded conductivities up to 0.01 S/cm [154]. Tigellar and coworkers reported conductivities of 0.05 S/cm at 150 °C using pyridinium-containing diamines as ILs [155]. Schmid-Naake and coworkers prepared composite membranes containing 1-hexyl-3-methyl-imidazolium/HMI-, 1-butyl-3-methyl-imidazolium/BMIM)-, and pyrrolidinium (1-butyl-1-methyl-pyrrolidinium/BMPyr)-based ionic liquids with hydrophobic anions such as tris(pentafluoroethyl)trifluorophosphate, bis(trifluoromethylsulfonyl)imide, hexafluorophosphate, and tetrafluoroborate. As expected, Nafion conductivity decreased with temperature, but the conductivity of the composite membranes with ILs increased with temperature up to 1 mS/cm [156]. As shown in the examples, ILs can be an alternative in PEMFCs operating under anhydrous conditions, but composite membranes did not show proper ionic conductivity, as observed for other fillers.

SPEEK membranes have also been doped with ILs to reach proton conductivities similar to those of Nafion membranes. In a recent work from Shahi and coworkers, the incorporation of 70 wt.% of 1-ethyl-3-methylimidazolium ethyl sulfate into SPEEK created membranes with proton conductivities up to 20 mS/cm at 150 °C under anhydrous conditions [157]. Polymerized imidazolium ionic liquids have also been used in increased proton conductivity in SPEEK membranes [158,159,160,161]. Various ionic liquids based on BMIM were used as fillers with enhancing effects on the properties of SPEEK-based membranes co-doped with mesoporous silica [162]. These composite membranes reached a conductivity of 15.0 mS/cm at 180 °C. In a recent review by Elwan, Mamlouk, and Scott, the use protic ionic liquid as fillers in polymer blends for polymer electrolyte membrane fuel cells was discussed in detail [163].

**Figure 9:**
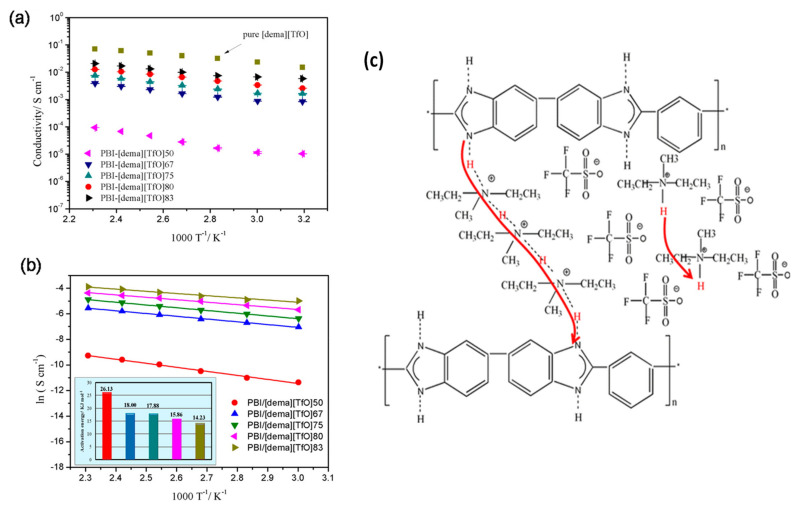
(**a**) Variation of the conductivity of the IL and membranes at different temperatures. (**b**) Curve of ln σ vs 1/T (inset picture: activation energy of different composite membranes). (**c**) Hypothesis of ion conduction in the PBI-based membrane. Reprinted with permission from reference [166]. Copyright 2014 American Chemical Society.

The combination of ionic liquids with polymeric membranes based on PBI polymers has afforded the preparation of composite membranes with proton conductivity values close to 0.1 S/cm. Representative examples of these membranes were reported by Wang and coworkers by using fluorine containing PBI and 1-hexyl-3-methylimidazolium trifluoromethanesulfonate as filler, reaching a proton conductivity of 0.016 S/cm at 250 °C under anhydrous conditions [164]. The use of 1-H-3-methylimidazolium bis(trifluoromethanesulfonyl)imide IL in a PBI membrane afforded a proton conductivity of 0.002 S/cm at 190 °C [165]. Liu et al. prepared membranes with diethylmethylammonium trifluoromethanesulfonate with a conductivity of 0.02 S/cm and a good performance as a H_2_/Cl_2_ fuel cell under anhydrous conditions (Figure 9) [166]. These last examples are referred to as undoped membranes, but PBI composite membranes containing ionic liquids can be used with acidic doping to reach higher conductivities as the ionic liquid can partially retain phosphoric acid, reducing its leaching from the membrane. Using this strategy, our group prepared PEMs based on PBI filled with 1-butyl-3-methylimidazolium (BMIM) and different anions, which created composite membranes with proton conductivities up to 0.1 S/cm at 120 °C [167,168]. A different type of PBI, such as poly(oxyphenylene benzimidazole) (OPBI), has also been employed for the preparation of composite membranes using BMIM derivatives, reaching good conductivities and high proton selectivity in vanadium redox flow batteries [169]. Other ionic liquids, such as the high Brønsted-acidic 2-sulfoethylmethylammonium triflate [170], 1-ethyl-3-methylimidazolium triflate, or 1-ethyl-3-methylimidazolium bis(trifluoromethanesulfonyl)imide [171], have also enhanced the proton conductivity of PBI membranes.

### 2.4. Nanofibers

Nanofibers constitute an elegant approach to reinforce polymeric membranes. One method is blending of the ionomer with a miscible polymer, which exhibits good stability in respect to mechanical stresses. Another method for reinforcing a membrane is the incorporation of a mechanically stable porous network of nanofibers by infiltration with the ionomer material. Regarding the first method, blends of polyvinylidene fluoride (PVDF) and Nafion have proven to reduce methanol and hydrogen crossover in direct methanol fuel cells (DMFCs) and PEMFCs [172,173], as well as to increase the tensile strength blend ionomers for proton exchange membrane fuel cells [174]. However, the second method has been found to be more beneficial in regards to the PEMFC lifetime compared to the blended composite membrane [175]. For the fabrication of a highly porous, interconnected reinforcement network, electrospinning is a popular technique.

Nanofibers constitute another class of commonly used fillers used in the fabrication of reinforced composite membranes to be used as PEMFCs, as these nanofibrous structured materials increase water retention and, consequently, the proton conductivity of the membrane [176]. Electrospinning has been progressively stablished as a versatile method for generating ultrathin nanofiber-based architectures [177]. The morphology and diameter of electrospun fibers can be tuned by controlling different parameters, which include the intrinsic properties of the solution, such as the type of polymer, viscosity, concentration, elasticity, and surface tension of the solvent, among others, but also the operational conditions, such as the electric field applied in the process, the distance between spinneret and collector, and the feeding rate for the polymeric solution.

The use of electrospun nanofibers in composite membranes of perfluorosulfonic acid polymers, such as Nafion, Fumion, and Aquivion, has been studied over the last 15 years [178]. In this regard, a Nafion film reinforced by poly(phenyl sulfone) nanofiber displayed similar proton conductivity to that of a pristine Nafion membrane, but with enhanced mechanical stability [179]. In a more dramatic example, uniaxially aligned sulfonated polyimide (SPI) nanofibers were prepared by the electrospinning method and displayed conductivities up to 7 S/cm at 90 °C and 95% RH, 30% RH. These SPI electrospun nanofibers were incorporated into Nafion membranes, increasing their proton conductivities above 1 S/cm. The high proton conductivity of Nafion nanofiber was also observed for pure Nafion nanofiber, which reached a value of 1.5 S/cm, being an order of magnitude higher than a pure Nafion film (0.1 S/cm) [180].

Conductivity and water uptake increase with sulfonation degree and the dependence of conductivity with water content were stronger for SPEEK materials than in the case of Nafion [181,182]. Under certain conditions of temperature and sulfonation degree, the conductivity of SPEEK at high hydration levels surpasses that of Nafion^®^ (~0.1 S/cm) [64,183]. As mentioned before, water uptake is a key parameter relevant to proton conductivity. Methods to control water uptake involve polymer blending [184,185,186,187,188] and crosslinking, which can be carried out chemically [189,190,191,192,193,194,195,196] or ionically [197,198]. Other authors have also reported the possibility for an additional self-crosslinking reaction in SPEEK via inter-chain polymerization of the sulfonic acid groups at high temperatures under vacuum [199]. A peculiar characteristic of the hydrocarbon-type membranes is the fact that their properties become dependent on the pre-treatment and thermal history, as well as on the solvent used for the membrane casting [62,63,200,201,202]. In this regard, dimethylformamide (DMF) [63,201,202] and dimethylsulphoxide (DMSO) [202] are reported to very negatively affect the performance of the membranes, while dimethylacetamide (DMAc) and N-methyl-2-pyrrolidone (NMP) solvents seem more appropriate to achieve better properties.

**Figure 10:**
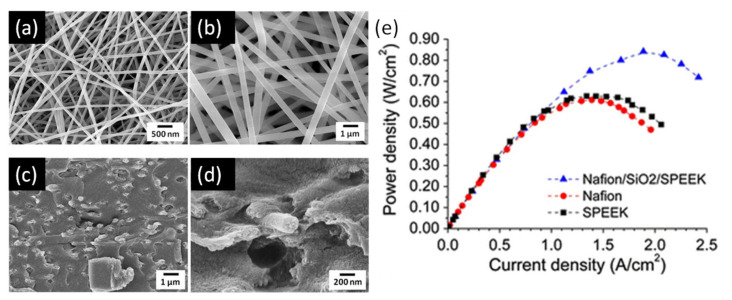
SEM images (surface view) of electrospun (**a**) SPEEK nanofiber mat (at 16 kV, 8 cm, 0.080 mL/h), (**b**) SEM images (cross-sectional view) of Nafion-impregnated electrospun SiO_2_/SPEEK (40/60 *w*/*w*) nanofiber composite membranes at (**c**) lower magnification (×10,000) and (**d**) higher magnification (×50,000). (**e**) Power density curves of the Nafion-impregnated SiO_2_/SPEEK composite nanofiber membrane, recast Nafion, and SPEEK film at a 75 °C and 100% RH. Reproduced by permission of Springer from reference [197].

SPEEK membranes can also be reinforced by using electrospun nanofibers and high performance in terms of proton conductivity has been described [203]. SPEEK combined with SiO_2_ was electrospun into nanofibers and composite PEMs were fabricated by impregnation with Nafion (Figure 10). The resulting PEMs displayed improved water retention and proton conductivities up to 0.08 S/cm at 90 °C and 100% RH [204]. Polarization curves of single cells with the Nafion-impregnated SiO2/SPEEK composite nanofiber membrane showed that the maximum power density of the nanofiber composite membrane was 170 mW/cm^2^ compared to 71 mW/cm^2^ for the pristine Nafion membrane.

**Figure 11:**
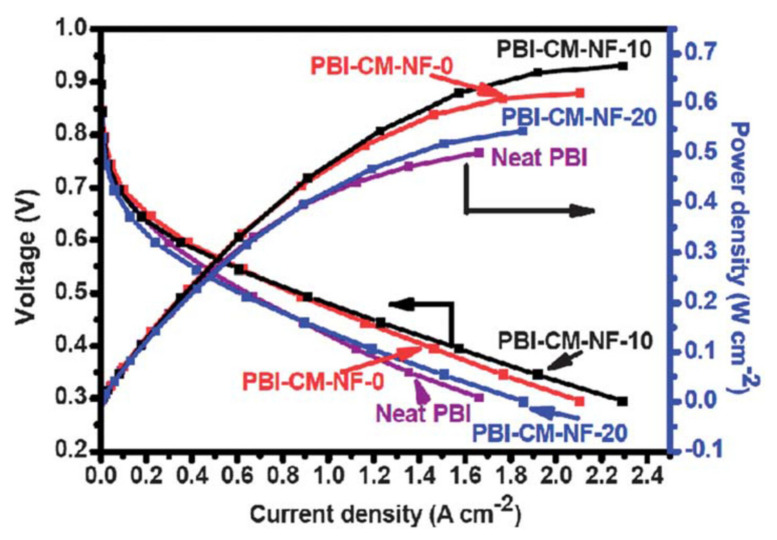
Single-cell tests (150 °C with a hydrogen–oxygen system) of the nanofiber-reinforced PBI composite membranes (PBI-CM-NF-X); the data recorded with the neat PBI membrane are included for comparison. Reproduced by permission of The Royal Society of Chemistry from reference [205].

Composite PBI-based PEMs containing nanofibers have also been fabricated and reinforced membranes with enhanced conductivity have been obtained. A PBI membrane containing polybenzoxazine nanofibers showed a significant enhancement of the mechanical properties, reaching conductivity values up of 0.2 S/cm at 160 °C after acid doping [205]. Single-cell tests showed a current density at 0.4 V of 1.55 A cm^−2^ for the PBI membrane with nanofibers (Figure 11). PBI composite membranes containing poly (aryl sulfone ether benzimidazole) nanofibers reached a proton conductivity level of 0.067 S/cm at 160 °C [206]. Silica nanofibers containing neutral, acidic, and basic groups have also been described as efficient fillers for composite PBI membranes [207]. Neutral nanofibers were fabricated through the electrospinning process, functionalized with acidic and basic groups by means of silane chemistry [208,209,210,211], and characterized by X-ray photoelectron spectroscopy (XPS) [212,213]. Proton conduction studies concluded that PBI composite membranes with basic nanofibers displayed an enhanced behavior over neutral or acidic nanofibers, displaying proton conductivities up to 0.004 S/cm at 200 °C.

## 3. Electrocatalysts and Electrodes

**Figure 12:**
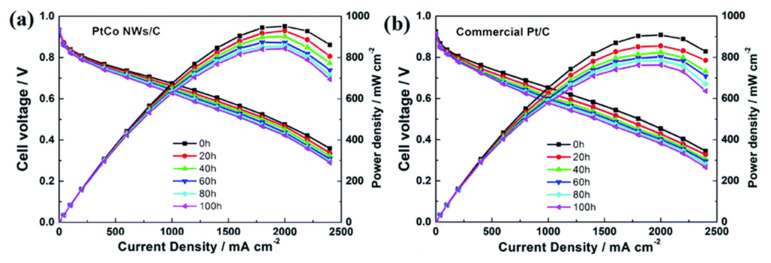
Polarization curves of single cell using (**a**) Pt–Co NWs/C and (**b**) commercial Pt/C under the 1000 mA cm^−2^ for 100 h. Reproduced from reference [214] with permission from the Royal Society of Chemistry.

In electrocatalysis reactivity, interfacial kinetics of active materials play an important role. The electron transfer between the electrode and deposited catalyst is in conjunction with several slower processes, such as adsorption of reactants and conversion to intermediates. The formation and desorption of products, as well as mass transport (i.e., molecular diffusion) between catalyst surface and solution, all of which are convoluted at or near the surface of the catalysts, lead to higher catalyst efficiency, higher catalyst stability, and better scalability [214] (Figure 12). Material properties and process modeling with density functional theory (DFT) represents an accurate method to facilitate the study and computation design of materials and for the development of different electrochemical fuel cell technologies [215,216,217], mainly to achieve alternative methods for energy conversion.

The kinetically sluggish cathodic oxygen reduction reaction (ORR) affects the massive development of PEMFCs. Therefore, the high performance of cathode electrocatalysts must be improved for solving this problem. Recent progress in precious-metal-free carbon-based materials [218,219] towards the oxygen reduction reaction have been reviewed, analyzing the enhancing catalytic activity, stability, and anti-poisoning presented on precious-metal-free carbon-based materials. They have been paid increasing attention for their unique electronic features, tunable nanostructures, and robustness, incorporating the latest strategies by increasing the accessible active sites and promoting the intrinsic activity of these catalysts. Among the various methods for fabricating nanoscale electrocatalysts, electrospray and electrospinning technologies are low-cost, facile, and industrially recommended routes to nanotechnology over the past ten years [220]. Besides, challenges and future prospects for the manufacturing of nanocatalyst technologies are analyzed in detail on this paper. In this direction, efficient synthetic methodology of Pt-Co nanowires as cathode catalysts for proton exchange membrane fuel cells have been fabricated and characterized [221], using a hexacarbonyl precursor to form nanowires that act as a reducing agent and as a structure-directing agent. Electrochemical performance in a half-cell test attained a mass activity of 291.4 mA mgPt^−1^, which is significantly better than the commercial carbon-supported Pt catalyst with 85.5 mA mgPt^−1^, where, after the accelerated durability test (ADT), the carbon-supported nano-alloyed catalyst showed an electrochemical active surface area (ECSA) loss of 19.1%, while the loss in the commercial catalyst was 41.8%. Results of this experimental development indicate that the fabricated one-dimensional structure is favorable to improve the catalytic activity and durability for PEMFC applications. To date, a major research effort has been devoted to significant developments of highly active and stable electrocatalysts in order to reduce the content of platinum group metals in PEMFCs through the combination of theory and experimental approaches [217].

### 3.1. Oxygen Reduction Catalysts

Single metal of platinum group metal (PGM), platinum group metal alloys, and single-atom-based metal oxides and metal nitrides are used like active catalysts for the oxygen reduction reaction (ORR) [222,223,224,225]. In literature, many reviews have recently reported [226,227,228] mentioning that some Pt-based alloy catalysts showed higher activity than Pt alone, and this has been attributed to changes in the Pt–Pt bond distance and Pt electronegativity [229], electron density in the *5d* Pt band [230], and surface oxide layers [231].

**Figure 13:**
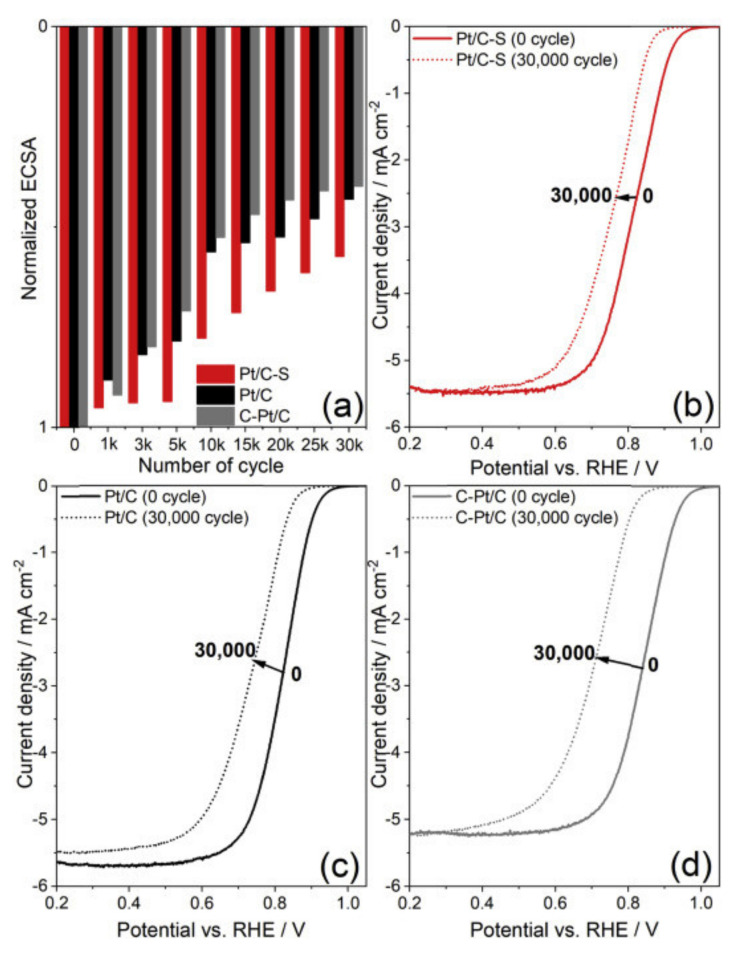
(**a**) Normalized ECSA until 30,000 cycles of the AST durability test in N_2_-saturated 0.1 M HClO_4_ of Pt/C-S, Pt/C, and C-Pt/C. ORR polarization curve before and after 30,000 cycles of AST of (**b**) Pt/C-S, (**c**) Pt/C, and (**d**) C-Pt/C. Numbers in (**b**–**d**) indicate the initial and final cycle of the AST durability test. Reproduced with permission from Elsevier (reference [235]).

Platinum is the most active metal catalyst for ORR of the entire periodic table; however, its use in bulk has been replaced by its use in the form of carbon-supported nanoparticles, which face, among other problems, important challenges in terms of stability and durability. There are different proposals to reduce the instability of the Pt/C, one of the first is to change the use of carbon black as the supporting material to another with well-defined structure such as CNT [232], nitrogen, and sulfur-doped CNT [233], or the use of N-doped graphene-TiO_2_ [234], those changes would enable avoiding problems related to corrosion of the carbon support. On the other hand, the work of Ham et al. [235] proposed the use of Pt nanoparticles covered by an S-doped carbon layer to accomplish a durable and active catalyst compared with Pt/C (Figure 13). However, Pt-based alloy catalysts show superior activity compared to Pt alone, attributed to changes in the Pt–Pt bond distance, Pt electronegativity, electron density in the *5d* Pt band, and surface oxide layers.

Another strategy to increase the support stability is by incorporating metal oxide as catalyst supports. For this reason, a wide variety of options for novel Pt supported with metal oxides, such as NbOx [236], In_2_O_3_ [237]_,_ and TiO_2_ [238]_,_ have been explored. However, those kinds of metal oxide materials have poor conductivity, which makes it necessary to develop different strategies to increase conductivity, such as:

- Adding carbon-based materials like CNT [232], C [236], or graphene nanoribbons [238];
- Alloying of Pt with other metals, such as In [237];
- Incorporating some nitride compounds, such as TiN [239].

**Figure 14:**
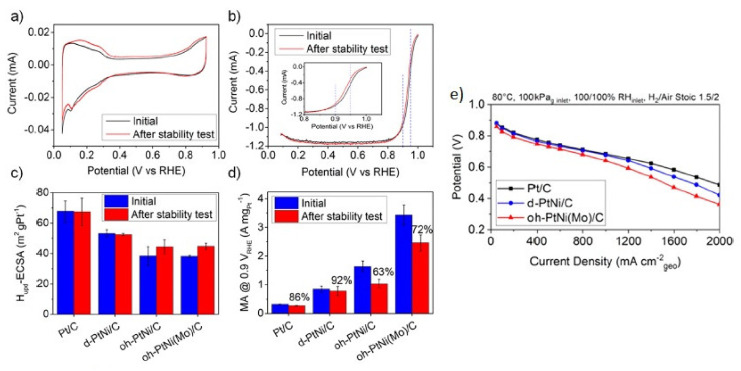
(**a**) Cyclic voltammetry oh-PtNi(Mo)/C at 20 mVs^−1^ and 0.1 M HClO_4_ saturated with N_2._ (**b**) LSV after activation (black) and after the stability test (red) for oh-PtNi(Mo)/C. CV and LSV were obtained at 20 mVs^−1^ and 0.1 M HClO_4_ saturated with O2 and 1600 rpm. (**c**) Hupd-ECSA after activation (blue) and after stability test (red) for Pt/C, d-PtNi/C, oh-PtNi/C, and oh-PtNi(Mo)/C. (**d**) Mass activity at 0.9 vs. RHE. (**e**) MEA single-cell performance with oh-PtNi(Mo)/C (red), d-PtNi/C (blue), and Pt/C (black) as the cathode and Pt/C as the anode in H_2_/air Reprinted with permission from reference [244]. Copyright 2016 American Chemical Society.

The above strategies result in conductive, stable, and active catalysts in half-cell experiments and in PEMFC tests. One of the first steps in the development of more active catalysts was to form Pt-based alloys, Pt-M type (M = transition metal, e.g., Pt-Au [240], Pt-Fe [241], Pt-Ni [242]) or Pt-MN (M ≠ N e.g., Pt_2_CuNi [243] or PtNiMo [244]). Yin et al. reported the production of Pt-Au alloy nanoparticles, which not only achieved a more active catalyst than Pt/C, but also explained the effect that the heat treatment has on the structure of nanoparticle, and how this treatment considerably increases the catalytic activity of the bimetallic material. The PtNi doped with Mo is one of the most active and stable catalysts and, in the work of Strasser et al. [244], they explained that molybdenum is the crucial element of the activity and stability of the Pt-Ni catalyst since Mo prevents the Ni segregation. However, a further step is the synthesis of alloys with well-defined geometric structures that allow the increase of the catalytic activity by optimizing the use of platinum on the electrode surface. This is a situation observed on the core–shell structures, i.e., PdM@Pt (M = Fe, Ni, and Co) type [245]. The effect of the increase on Pt alloys’ catalytic activity is well studied and is assigned to a decrease in the oxygen adsorption energy on the surface in a range of 0.2 eV. Figure 14 shows different studies of cyclic voltammetry for Pt/C, d-PtNi/C, oh-PtNi/C, and oh-PtNi(Mo)/C.

There is a new variety of carbon-based catalysts (M–N–C type) that have a metallic atom (Mg, Ca, and Al [246]; Fe, Co, Cu, Zn, and Ru [247,248]; and Ir [249]) coordinated with different numbers of pyridine nitrogen atoms confined in a graphene matrix. The structure is inspired by cofactors presented in enzymes, which are usually biochemically active. Some of its outstanding features are the high surface area of the carbon base structure that allows it to distribute and expose the active sites efficiently; good pore size of the support, which enables good mass transfer; and graphitic structure with good electrical conductivity and good distribution of catalytic sites [250]. However, those kinds of catalysts face some challenges, such as their low stability and poor durability. The amount of catalysts required for a PEM cell is much higher than for metallic catalysts, which could cause difficulty in handling of the cells, and their catalytic activities are not as good as those of platinum alloys. These materials need to be catalytically improved in order to represent an alternative for elimination of Pt-based catalysts used in the cathodic site of a PEM fuel cell.

**Figure 15:**
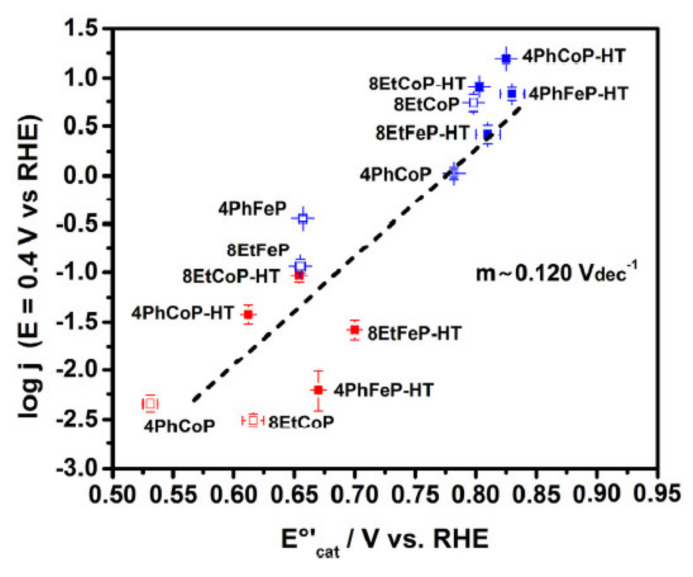
Plot of activity expressed as log_(j)_ at E = 0.4 V (vs. RHE) as a function of the redox formal potential (E°) of the studied intact (open symbols) and heat-treated (closed symbols) Fe- and Co-based catalysts. The dashed line shows the obtained linear trend. Reproduced with permission from Elsevier (reference [250]).

**Figure 16:**
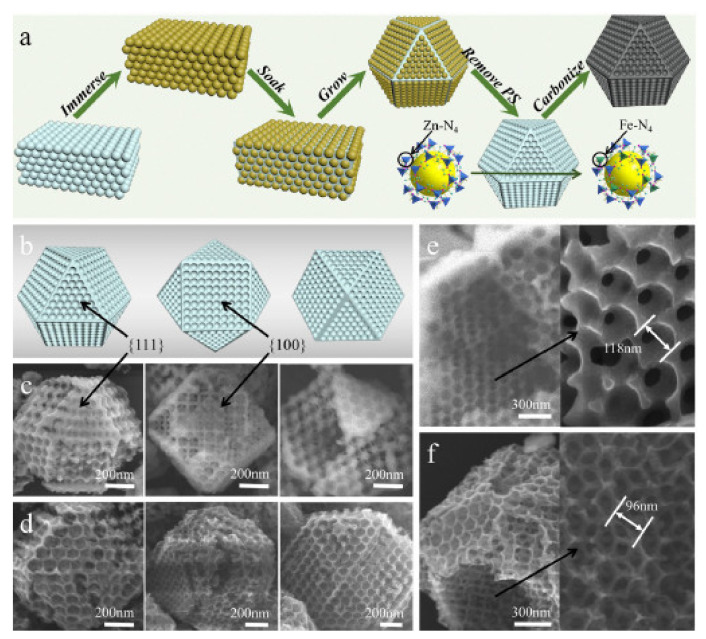
(**a**) Schematic illustration of the synthesis of FeN_4_/HOPC-c-1000. (**b**) Models of OMS-Fe-ZIF-8 viewed from different directions. (**c**) SEM images of OMS-Fe-ZIF-8. (**d**) SEM images of FeN_4_/HOPC-c-1000. (**e**) SEM images of broken OMS-Fe-ZIF-8 crystal. (**f**) SEM images of broken FeN_4_/HOPC-c-1000 particle. Reproduced by permission of John Wiley & Sons, Inc. from reference [251].

This new M-N-C type is still being studied to understand its kinetic ORR mechanism [248], associated with the metal center concentration [250], its reduction potential [251], and its catalytic activity. In this sense, Venegas et al. found a linear relationship between redox potential and catalytic activity (Figure 15). The observed trend represents a descriptor of catalytic activity, as with more positive redox potential, greater is the catalyst activity. Among different metal centers where ORR is carried out, catalysts with iron atoms are the most widely investigated [252,253,254] due to their good catalytic activity, relatively good chemical stability, the formation by multi-electron charge transfer (n = 4e^−^) to water formation, and low cost of chemical precursors used in the chemical synthesis (Figure 16). However, there is evidence that indicates that the ORR on electrode surfaces containing iron atoms degrades the polymeric membrane via the Fenton reaction, which produces H_2_O_2_ and is the reason the cobalt compounds are beginning to gain importance [248].

**Figure 17:**
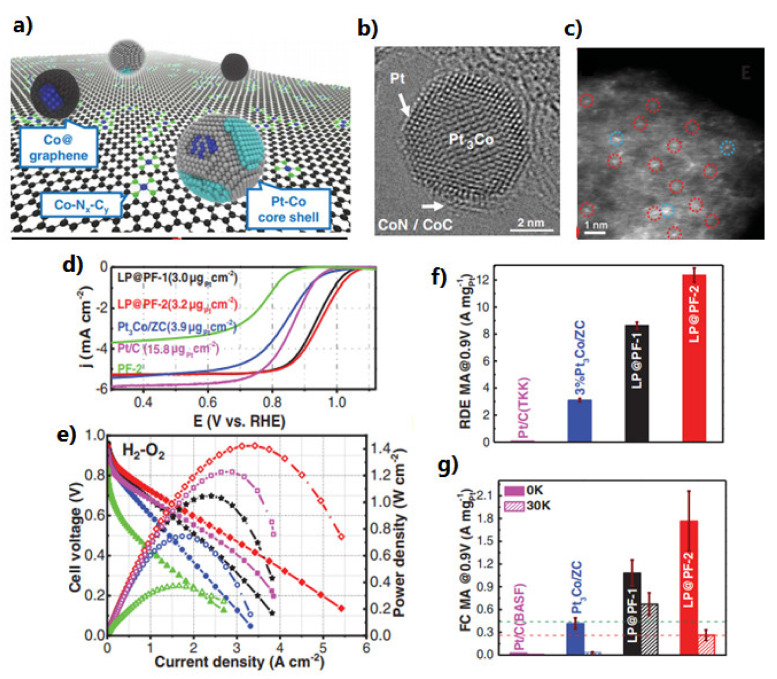
(**a**) Schematics of LP@PF catalysts, showing coexistence of Pt-Co NPs, Co@graphene, and Co-Nx-Cy PGM-free active sites. (**b**) HAADF-STEM image of Pt-Co NPs in LP@PF-1. (**c**) PGM-free support containing atomically dispersed Co (circled in red) and trace Pt (circled in blue). (**d**) LSVs of different catalysts recorded at a rate of 10 mV s^−1^ and 1600 rotations per minute in O_2_-saturated 0.1 M HClO_4_; j, current density. (**e**) H_2_-O_2_ fuel cell i-V polarization (solid symbols and lines) and power density (hollow symbols and dashed lines) plots recorded under 1 bar of O_2_ pressure with cathode Pt loading of 0.033 mg_Pt_ cm^−2^ for LP@PF-1 (black stars), 0.035 mg Pt cm^−2^ for LP@PF-2 (red diamonds), 0.043 mg_Pt_ cm^−2^ for Pt_3_Co/ZC (blue spheres), and 0.35 mg_Pt_ cm^−2^ for commercial MEA (magenta squares); PF-2, green triangles. (**f**) Comparison of MAs at 0.9 V versus RHE. (**g**) Fuel cell (FC) MAs at 0.9 V iR-free before (solid) and after (hatched) 30,000 voltage cycles, showing that LP@PF catalysts meet or exceed DOE’s 2025 MA targets for before (green dashed line, 0.44 A mgPt^−1^) and after (red dashed line, 0.264 A mgPt^−1^ or 40% of the initial value) AST. [255]. Reprinted with permission from AAAS.

In this regard, Chong et al. developed a catalyst consisting of Pt-Co nanoparticles (Figure 17a,b) supported on a Co-Nx-C-type structure (Figure 17c), demonstrating an enhanced catalytic activity and record stability (Figure 13g), superior to Pt/C in half-cell (Figure 17d,f) and in a PEMFC (Figure 17e), exceeding the values established by DOE for performance catalysts [255].

**Figure 18:**
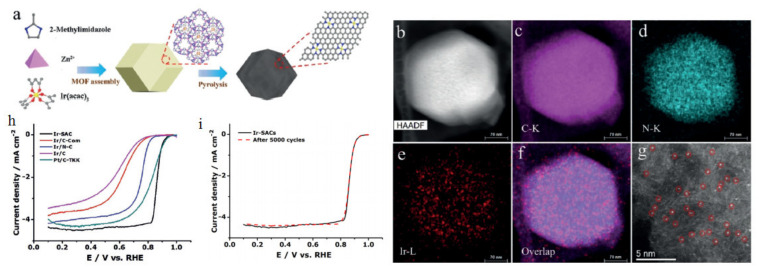
(**a**) Scheme of the fabrication of single atomic site catalysts, (**b**–**f**) STEM images and the corresponding elemental mappings for the Ir-SAC, (**g**) high-resolution HAADF-STEM image of Ir-SAC, with the distinct bright dots (circled in red) indicating Ir is atomically dispersed on the nitrogen doped carbon matrix. (**h**) ORR polarization curves with a scanning rate of 5 m Vs@1 at rotating speed of 900 rpm for the synthesized catalysts. (**i**) ORR polarization plots of Ir-SAC before and after potential cycling stability tests. Reproduced by permission of John Wiley & Sons, Inc. from reference [249].

Another catalyst with remarkable catalytic activity is that obtained on Ir-N_4_-C (Ir-SAC). Xiao et al. [249] synthesized a single atom catalyst (SAC) by in situ impregnation of Ir (Figure 18a), the result of which was electrochemically more active than the Ir/C nanoparticles (Figure 11). This behavior was associated with the configuration of Ir-N_4_ (Figure 18b–g), which allows an optimal oxygen absorption energy for ORR (as demonstrated using DFT). In addition, the excellent anchoring of the Ir atoms to the nitrogen atoms allows an outstanding stability compared to Pt/C (Figure 18i), without Ir atoms’ dissolution on the electrode surface.

For the synthesis of these new materials, there is a wide variety of methodologies that allow for obtaining nanostructured carbon matrices with high surface areas decorated with transition metal species, such as the templated method, the self-templated method, the impregnation process, mixture approach, leaching method, and the combination of several methods denoted as multiprocess [248]. Less conventional methods are ball milling and the ionothermal. Another possibility is to obtain M-N-C materials through an organic framework metal synthesis. In that sense, Lu et al. summarized the four main MOF synthesis strategies as: (1) direct pyrolysis of pristine MOFs, (2) pyrolysis of MOFs with space-confined metal-containing molecules, (3) pyrolysis followed with an acidic wash, (4) top-down synthesis from bulk metals [256].

On the other hand, regarding transition-metal oxides, Song’s group [257] were the first to report a transition-metal oxide without C hybridization for ORR in an acid medium, contrary to what one would think that catalysts with conductivity problems are, due to the nature of metal oxide, which contains water molecules in its structure (WO_3_·2H_2_O), allowing significant improvement in its conductivity. This is in addition to the fact that the presence of oxygen vacancies in the structure of WO_3_ give it catalytic activity towards ORR. However, the path in the investigation of these materials is just beginning, and that is why they still present important challenges related to their low catalytic activity compared to Pt/C.

Titanium oxynitrides are another type of material whose catalytic activity originates from oxygen vacancies [258]. These materials have shown that the catalytic activity not only depends on the number of N atoms bound to the metal atom of the catalyst, but also strongly depends on the number of oxygen vacancies present in the catalyst. Researchers found that at higher synthesis temperature, a more significant number of vacancies and a superior catalytic activity were produced.

The bimetallic nitrides ML-N (M ≠ L; M = Ti, Ni, Co, and Mo) [259,260,261] are a group of catalysts little studied for ORR. Among the most recently analyzed, titanium nitride doped with Co [262] showed that by incorporating Co in its structure, the catalytic activity increased significantly. However, Tian et al. did not explain why the concentration of 20% of Co is the optimal for that catalyst, nor how the incorporation of cobalt modifies the reduction reaction mechanism, changing from via n = 2e^−^ for TiN to multielectron transfer n = 4e^−^ for Ti_0.8_Co_0.2_N. Besides, even the catalyst with the optimized cobalt concentration has a catalytic activity very far from Pt/C in an acid medium. On the other hand, Khalifah’s group [261] analyzed the Co0.6Mo1.4N, finding that hexagonal molybdenum nitride structure improved the catalytic activity via the presence of Mo^3+^ presented in octahedral coordination.

### 3.2. Hydrogen Oxidation Catalysts

The hydrogen oxidation reaction (HOR) on a Pt-based catalyst in a PEMFC is a fast process (two orders of magnitude compared to alkaline medium, in an alkaline fuel cell), in which the hydrogen gas is adsorbed on the catalytic surface, followed by the H-H bond breaking, and finally desorbed from the surface like a proton (being the rate-limiting step) [263,264]. The process’ speed is mainly attributed to the very fast transport of the H^+^ in water and the weak bonding energy in hydrated protons (H_2_O-H^+^) involved in the reaction [265].

Despite being a reaction with fast kinetics, there is prevailing interest in the reaction mechanism and if it is modified with pH. In this sense, important trends have been found, such as the fact that with an increase in pH value (increase in the hydrogen binding energy), a decrease in catalytic activity occurs. Therefore, the idea of using hydrogen binding energy as the only descriptor of catalytic activity for HOR in density functional theory (DFT) was well accepted only in some cases [266]. However, recently, the effect of interfacial water in the HOR kinetics at different pH values was evaluated [264]. In that work, the catalytic activities of Pt (111) and Pt (110) in acid and basic pH levels were compared, finding that in the acid medium, the HOR on Pt (110) was faster than on Pt (111). This behavior is attributed to the ion diffusion and to the structure of interfacial water molecules, although this does not play a relevant role in the kinetics of the reaction.

**Figure 19:**
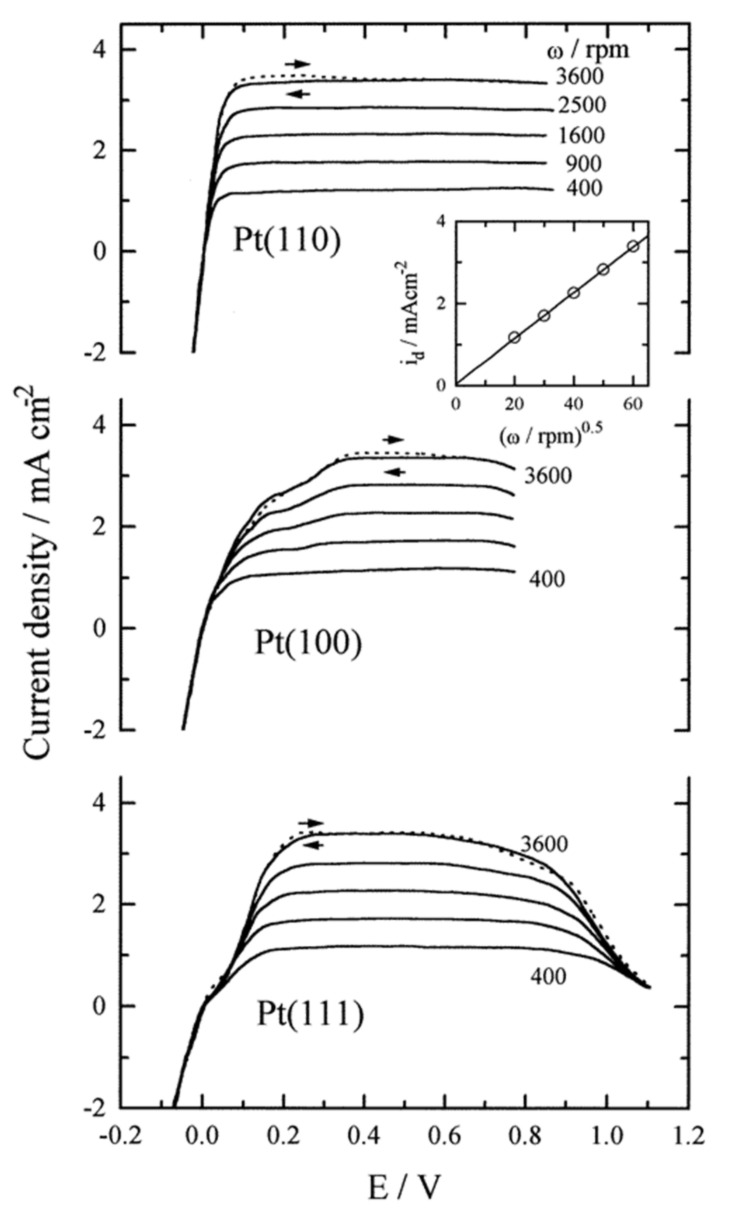
Polarization curves the HOR on Pt(hkl) in 0.05 M H_2_SO_4_ at 274 K; 10 mV/s. Reprinted with permission from reference [267]. Copyright 1997 American Chemical Society.

The pioneers in the analysis of HOR on the different crystallographic planes of Pt in an acid medium were Markovic et al. [267]. They gave rise to the following activity trend Pt (110) > Pt (100) > Pt (111) (Figure 19), where Pt (110) has an atom–atom recombination step (Tafel mechanism) as the rate-determining step (rds); Pt (100), has an ion–atom reaction (Heyrovski), which controls the rate of the reaction; and in Pt (111) the rds was unsolved [267].

Another parameter whose behavior is interesting to know is how Pt particle size affects the catalytic activity of HOR. Contrary to the particle size’s behavior for ORR, for HOR the smaller the particle size, the greater the catalytic activity [268]. That assertion agrees with what was previously discussed about the studies of a single crystal of Pt (110) [264], since those planes are directly associated with edges and are dominant in nanoparticles when particle size decreases close to 1.8 nm. Besides, it is also possible to observe the trend that the smaller the particle size, the lower the hydrogen bonding energy, and, as mentioned previously, the higher the catalytic activity. In contrast, in large cuboctahedral nanoparticles (~10 nm), where Pt (111) facets are four times more frequent than Pt (100) and the edges only represent 10% of the surface, the catalytic activity for HOR is minimal. That statement also explains why platinum nanoparticles are less active in an alkaline medium, since the edges have a greater affinity to adsorb anions and block the active sites of the catalyst than the facets [268].

**Figure 20:**
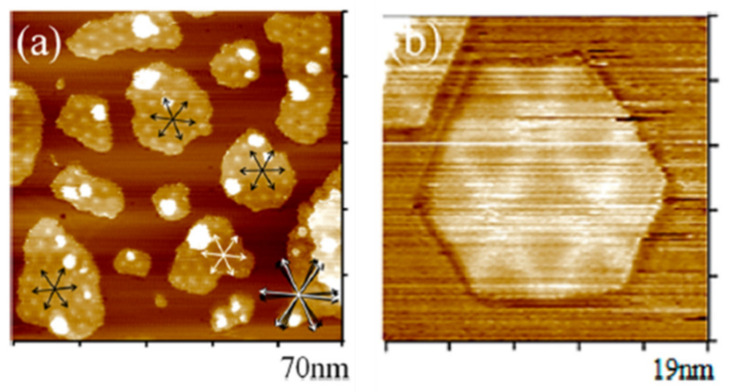
(**a**) Moire structure (M-phase) of Pt over Au (111), (**b**) Pt islands of Pt with well-defined hexagonal structure (R-phase). Reprinted with permission from reference [269]. Copyright 2016 American Chemical Society.

On the other hand, how the electrocatalyst’s structure affects the HOR activity was analyzed by Liao et al. [269], who evaluated the catalytic activity of a Pt monolayer deposited on an Au (111) substrate. Moreover, they found two relevant structures, well-defined hexagons (R-phase) and Moire structures (M-phase) (Figure 20). Of these two structures, the well-defined hexagonal structures were more active, and they attributed the improvement of activity to a more significant number of intrinsic active sites of the structure. Unfortunately, associating the catalytic activity with that alone can be tricky since, in this study, they initially assumed that the Au (111) substrate did not participate in the reaction, and this is not necessarily true. However, later they mentioned a modification in the Pt-Pt distance (5.5% larger in R-phase) that modifies the absorption energy of H and, therefore, causes an impact on the catalytic activity that could be caused by the “spillover” effect caused by the substrate.

Otherwise, the catalyst most widely used like an anode is platinum, and significate overpotential has not been reported when low loads of Pt/C are used (0.03–0.05 mg cm^−2^) [270,271]. For that reason, there is a much less marked tendency to investigate new platinum-based catalysts for HOR. Nevertheless, regarding the study of monometallic catalysts, Gasteiger et al. found that for monometallic nanoparticle catalysts, the exchange current density (i_o_) follows the trend Pt > Ir >> Rh > Pd when characterized using an H_2_ pump configuration [263].

Regarding platinum-based alloys, Gasteiger et al. [272,273,274] reported in 1995 the first study of Pt-Ru and Pt_3_Sn as HOR catalysts. They showed that both the mechanism and the speed of the reaction are very similar to that of carbon-supported Pt/C [272,273,274]. Another work related to Pt alloy appeared in 1999, and evaluated Pt-Ru and Pt-Sn alloys, but, unfortunately, both alloys showed activities well below Pt/C [275]. Subsequently, the study of Pt-Mn, Pt-Pb, Pt-Sb, and Pt-Sn found that only for the Pt-Pb alloy was the HOR not controlled by diffusion but by charge transfer. The catalysts analyzed followed catalytic activity trend Pt-Sb >> Pt-Sn > Pt >> Pt-Mn >> Pt-Pb (Table 1), and in a general way, it was found that the transition metals significantly improved the adsorption steps on the catalytic surface and this was the determining step of the reaction [276].

**Figure 21:**
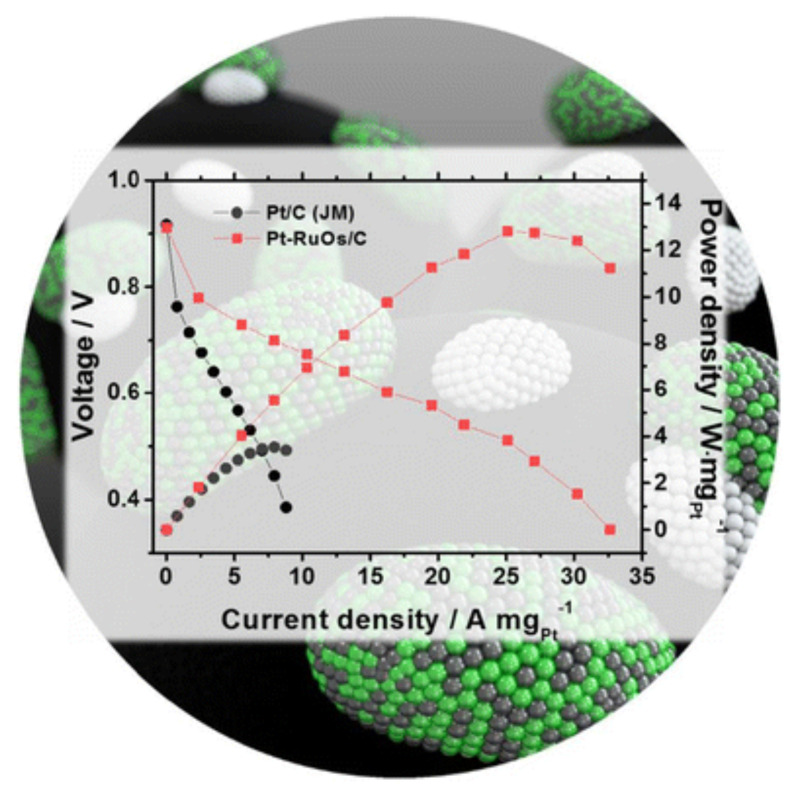
Performance of carbon-supported Pt/C and multicomponent low-Pt content Pt-RuOs/C. Reproduced with permission from Springer (reference [277]).

While in multicomponent catalysts, such as RuOs/C binary electrocatalysts and Pt-modified RuOs/C (Pt-RuOs/C) ternary electrocatalysts, a significantly enhanced electrochemical performance as an anode electrode (Figure 21) was exhibited for HOR in PEM fuel cells [277]. Results of this study demonstrated the use of multicomponent materials without Pt or with a very low Pt content as potential alternative anode catalysts in fuel cell devices.

On the other hand, several Pd-, Pt-, and Pt/Pd-based metallic glasses have been evaluated [278], showing promising performances and large electrochemical surface areas, attributed to their relatively lower work function. Analogously to what happens in ORR catalysts, various strategies seek to improve platinum’s catalytic activity and stability. One of the most common is to enhance the materials used as support for platinum nanoparticles, e.g., TiOx [279] and WC/C [280]. In this sense, Pt/TiOx/C systems have been proposed, where TiOx serves as a support material and is used to encapsulate platinum nanoparticles, and thus improves the selectivity of HOR since the TiOx layers are permeable to protons but not to hydroxyl ions. This system naturally incorporates carbon as support in order to avoid facing low-conductivity problems [279]. At the same time, the use of WC/C has been proposed to make the catalyst tolerant to the CO as an impurity present in the H_2_ when it is obtained from reformed fuels. This effect is produced by the tungsten carbides, in which the bonding between CO and tungsten carbides is much weaker than the bonding between CO and Pt.

Although excellent Pt-based catalysts have been developed for HOR, there is still a concern to decrease platinum use or eliminate it entirely from the anode electrodes. For this reason, the use of other noble metals, such as Pd and Ir in mono-metallic nanoparticles, as well as their alloys (Pd-Ir [281]; Pd-Co [282]; Pd_3_Co [283]; PdP_2_ [284]; Ir-M, M = Fe, Ni, Co [285]; Pd_3_P, IrP_2_, RuP, and Rh_2_P [286]), have been explored.

As it happens in the catalysts for ORR, it has been found that the incorporation of a second metal, such as Ir, Co, Fe, Ni, etc., can modify both the electronic structure and the bond distances of the base metal. Just as Pd or Ir modify the Pd-H_ads_ or Ir-H_ads_ energy interactions [285], in this way they improve the catalytic activity [284] and the stability [287] (Table 2).

Among noble metal-based catalysts, some combine the benefits of bimetallic catalysts with the improved properties of some alternative supports, such as partially exfoliated carbon nanotubes (Pd_3_Co/PCNT) [286], graphitic carbon nitride (Pd-Co/gCN) [285], reduced graphene oxide (Pd_3_P/rGO, IrP_2_/rGO, RuP/rGO and Rh_2_P/rGO) [288], and nitrogen-doped carbon (Rh-Rh_2_O_3_) [287] (Table 3).

One interesting example of that strategy is that reported by Chandran et al. [286], where they used Pd_3_Co supported onto PCNT for the HOR. They evaluated their catalyst as an anode in PEMFC and achieved better activity from Pd_3_Co over pristine carbon nanotubes (and other Pd-based catalysts). Moreover, they attributed the enhancement in the catalytic activity to the synergistic effect of one-dimensional CNT and two-dimensional graphene, this last result obtained by the impact of the oxidative treatment.

**Figure 22:**
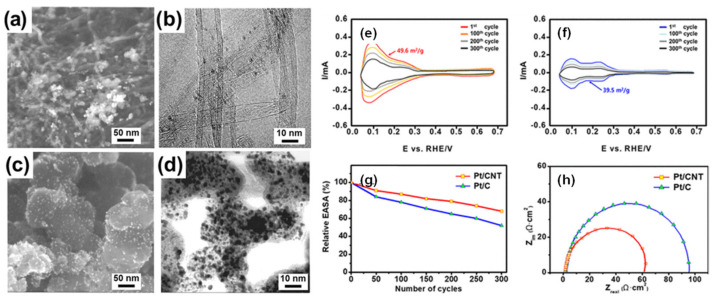
Morphological analysis using the SEM and TEM images of Pt/CNT (**a**,**b**) and Pt/C (**c**,**d**). Electrochemical performance of the electrocatalysts: (**e**) cyclic voltammograms of Pt/CNT and (**f**) Pt/C in an acidic medium (0.5 M H_2_SO_4_), (**g**) relationship between the normalized EASA as a function of the cycle number in an acidic medium (at +0.75 V vs. reversible hydrogen electrode (RHE)), and (**h**) Nyquist plots of the electrocatalysts. Reprinted with permission from reference [283]. Copyright 2016 American Chemical Society.

Likewise, the Pt/CNT catalyst had a larger active area than the carbon black supported Pt (Pt/C) catalyst and exhibited improved performance due to its long-term stability. The improved electrochemical performance and hydrogen oxidation reaction (HOR) mechanism of platinum loaded on a carbon nanotube (Pt/CNT) catalyst are reported, theoretical and experimentally supporting the research on the substrate effect of platinum-decorated carbon on enhanced hydrogen oxidation in PEMFC [289]. The charge–transfer resistance of Pt/CNT (61.2 Ω cm2) is much smaller than that of Pt/C (90.2 Ω cm2), indicating that the CNT support offers good electron transfer (Figure 22).

Although alternative catalysts to Pt have been synthesized, most of these still use noble metals such as Pd and Ir, with a tendency to be expensive. Unfortunately, there is limited interest in developing active and stable platinum-group-metal-(PGM)-free catalysts for HOR in PEMFC. In that sense, platinum-group-metal-(PGM)-free catalysts like TaSi_2_, MoSi_2_, Ni_2_Si, WSi_2_, WC, and WC (5 wt% Co) were evaluated without catalytic activity to HOR [288]. In contrast, W_2_C nanoparticles encased in N, P-doped few-layer carbon materials (W_2_C@N, P-C, denominated WNPC) showed an enhanced activity (1.03 mAcm^−2^), stability (10,000 cycles of accelerated degradation tests), and CO tolerance (1000 ppm CO/H_2_) originated by the uniform structure of WNPC, high electrical conductivity, large specific surface area, and the synergistic effect among N, P, and C [290].

**Figure 23:**
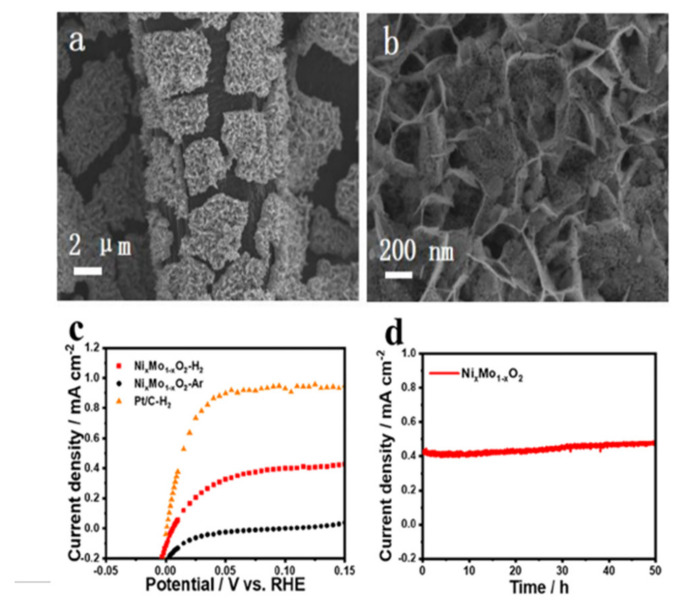
(**a**,**b**) Micrographs showing the structure of Ni_0.35_Mo_0.65_O_2_. (**c**) Steady-state polarization curves of Ni_0.35_Mo_0.65_O_2_ and Pt/C for HOR in 0.1 M HClO_4_. (**d**) Chronoamperometry curve in 0.1 M HClO_4_ at 0.1 V vs RHE. Reprinted with permission from reference [291]. Copyright 2016 American Chemical Society.

In the same field, the use of molybdenum dioxide (MoO_2_) has been carefully analyzed because it is chemically stable in acid medium. However, that material presents inferior activity toward HOR because it presents very weak adsorption of hydrogen atoms [291] and adequate energy adsorption is an essential feature in the PEMFC electrocatalysts [292]. Therefore, in the same way that the electrocatalysts for ORR are improved by increasing the active surface area or by adding a second metal to alter their electronic properties (electronic density), incorporating Ni into the MoO_2_ (Figure 23) can modify the adsorption energy of H_2_ atoms [291]. In that case, it is interesting that the Ni atoms that replace some Mo atoms do not change the crystalline structure of the MoO_2_. Furthermore, they produce an electron deficiency in the O sites, caused by higher electronegativity of Ni compared to Mo, increasing both the adsorption energy and the surface hydrogen coverage (40–50%) of the H_2_ atoms over the oxygen atoms of the catalyst, and the resulting Ni_0.35_Mo_0.65_O_2_ has a noticeable increase in the catalytic activity (~0.3 mAcm^−2^) with excellent stability, evaluated by chronoamperometry for 50 h.

Finally, a new group of materials has been used as anodes, including bioinspired nickel bis-diphosphine, supported on different unconventional carbon materials, i.e., CNT [293] and graphene acid [294], which are beginning to gain attention. However, they need to be studied carefully to assess their feasibility.

## 4. Membrane–Electrode Assembly (MEA): Preparation and Characterization

The catalyst layer is the key component of membrane–electrode assembly (MEA), as it has a major impact on the cell performance, stability, and lifetime durability, and represents the heart of hydrogen-powered PEM fuel cells. The exploitation of developing superior active and stable electrocatalysts has been the most promising strategy, followed by increasing the number of active sites and/or increasing the intrinsic activity to improve the performance of fuel cells [295]. The MEA preparation is extensively investigated for proper components optimization, preparing the catalysts blended with the appropriate ultrapure water, Nafion solution, and isopropanol in large-scale powertrain automotive and green technology applications [296]. The catalyst layer can provide active sites for the electrochemical reaction, as well as channels for proton, electron, gas, and water transport. Important aspects, such as the content of liquid Nafion and the characteristics of the solvent and additive used critically effect the MEA performance. Therefore, all the parts and components influence on the cost and properties of PEMFC. Important effects of the content of Nafion and the characteristics of solvent and additive used on the preparation of the ink-type electrodes, structures, and performances of MEA are currently being researched in order to reduce the content of platinum group metals (PGM) in electrodes, and, thus, the cost of hydrogen-powered cell vehicles. The progress in developing high proton conductivity and excellent mechanical stability under humid conditions with perfluorinated sulfonic acid (PFSA) ionomers have been reported [297], where long side chain (LSC) and short side (SSC) chain PFSA ionomer solid electrolyte membranes in the MEA preparation, tested by dynamic load-cycling, showed different behavior in short- and long-term applications with high stability with SSC. The tendency now is to evaluate in-line the direct coating processes for MEA preparation. These processes can efficiently produce MEAs at high speed, at low cost, and at high volume. In this context, significant progress and improvement has been made via the decal transfer method [298,299,300] for MEA preparation, with the advantage that the method presents homogenous coating of a porous transport layer, approaching a commercial reality in cost, break-in time, and cost for automotive transportation applications. However, as mentioned in this report, there remain two primary technical challenges to be addressed in the MEA. First and the foremost, is meeting the automotive cost targets: producing a fuel cell stack cost competitive with today’s internal combustion engine, where the stack assembly production methods must be amenable for use in low-cost, high-speed, automotive assembly lines. The second challenge is to achieve stability and longtime durability targets in real-world automotive duty cycle operations. Several articles were published recently that focused on fabrication of MEA samples via a decal transfer method [296], which used an ultrahigh-speed dispersion method to mix the catalyst ink, sprayed on a Nafion membrane at 80 °C to form the catalyst layer or deposited it by ultrasonic spray system, and then characterized this by electrochemical polarization curves and electrochemical impedance spectroscopy [301].

The structural features and construction methods of nanostructured MEAs based on one-dimensional nanostructures, such as nanowires, nanotubes and nanofibers, have been recently reviewed [302]. An overview on advances in fabricating ordered nanostructured MEAs with three-dimensional nanostructures based on different types of materials is presented and some perspectives on current limitations and future research directions of nanostructured MEAs are proposed. The effects of an ultrasonic dispersing methodology and time and cathode catalyst agglomerate size in PEMFC catalyst ink dispersions have been studied [303]. The cathode catalyst inks were characterized in order to elucidate the influences of the ultrasonic dispersing method and time on catalyst ink particle size and cathode catalyst layer formation. In situ ultra-small-, small-, and wide-angle X-ray scattering (USAXS-SAXS-WAXS) analyses were used to study the impact of ultra-sonication time and methodology on changes in the agglomerate, aggregate, and particle size and distribution during the dispersing process. Experimentally, it was found that a combination of brief tip sonication followed by bath sonication is the most effective at breaking up agglomerates, leading to maximum catalyst activity and MEA performance. It was reported that extended tip sonication was aggressive, inducing the detachment of the platinum nanoparticles from the carbon black support, which decreased the electrochemical surface area and MEA performance. Conditions such as 10 s tip followed of 20 min bath sonication was demonstrated to be an efficient way to break down carbon agglomerates into primary particles without separating the Pt nanoparticles. A combination of brief tip sonication followed by bath sonication was most effective at breaking up agglomerates, leading to maximum catalytic activity and MEA performance, as has been reported for developing outstanding performance of cathode electrodes through development and optimization of catalyst materials, electrode structure, and MEA preparation and characterizations [304,305,306,307,308,309]. Anisotropy of carbon paper microstructures can greatly affect the water droplet behavior through the porous gas diffusion layers [310]. Innovative concepts for improving the performance of membrane–electrode assemblies include using a dry-spraying preparation methodology, which is a time- and cost-effective method that involves solvent-free spraying of catalyst powder on the polymer electrolyte membrane [311]. This study demonstrated a pathway and methodology to improve MEA performance by optimizing ionomer composition, structure, and networks in the catalytic layer. The electrophoretic deposition (EPD) method, with considerable results in fabricating the gas diffusion electrode (GDE) in the absence of the binder for membrane–electrode assemblies (MEAs) in PEMFC operating at 140 °C, is presented in this article [312].

## 5. Performance of Single Monocells—PEMFCs

**Figure 24:**
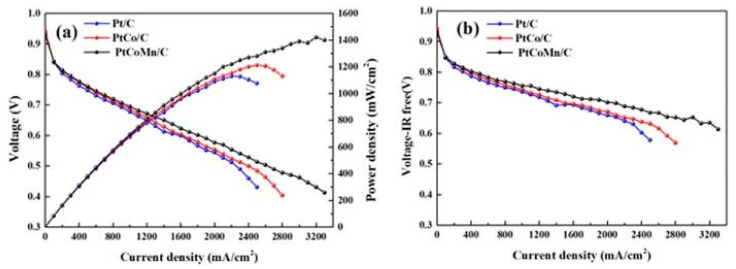
Polarization curves before and after IR free of MEA-PtCoMn/C MEA-PtCo/C and MEA-Pt/C: (**a**) original, (**b**) after IR free. [320] Reproduced with permission of IOPScience.

The exploitation of state-of-the-art of carbon-supported Pt electrocatalysts for PEMFCs is mostly limited, due to high Pt loading and durability issues caused by electrochemical instability by oxide formation on the carbon support in high-potential regimes. Hwang et al. reported [313] that high-compressive 3D Pt nanostructured thin films considerably increase the catalytic activity and electrochemical durability of electrocatalysts under PEMFC operating conditions. The nanostructure fabrication relies on the dealloying or selective leaching of solid alloys of Pt-C binary film to produce a residual 3D nanoporous thin film structure. Computational modeling has played a key role in advancing the performance and durability of PEMFCs. In recent years, there has been a significant focus on PEMFC catalyst layers due to their determining impact on cost and durability [314,315]. The catalyst layer poses many challenges from a modeling standpoint: it consists of a complex, multi-phase, nanostructured porous material that is difficult to characterize; it also hosts an array of coupled transport phenomena, including flow of gases, liquid water, and heat and charges, occurring in conjunction with electrochemical reactions. Strategies for multiscale simulations of catalyst layers that can bridge the gap between macroscopic and microscopic models, including modeling of liquid water transport in the catalyst layer and its implications on the overall transport properties, are included in the analysis. In an energy conversion stack, an essential component is bipolar plates (BPs) composed of metal-based materials, which can contribute to reaction gases, collect currents, remove product water, and cool the stack with sustainability, durability, and longevity. They have several issues to fulfill during PEMFC stack operation, and there are many challenges when it comes to metal-based BP materials. Carbon-based coatings have attracted considerable attention from both academia and industry, owing to their merits of high performance and low cost. For that reason, Yi et al. [316], presented a comprehensive survey considering recent progress in carbon-based coatings in terms of evaluation methods, material design, deposition process, and coating performance. Long-term durability is another challenge that can be addressed through the advancement of inexpensive and lightweight current collectors and highly efficient gas diffusion electrodes for better distribution of the reactants while maintaining an optimum hydration of the proton-conducting membrane. Finding suitable metal and alloy materials represents a significant task for BP materials, because they should have multiple qualities that sometimes come at the expense of one another. As BPs constitute a significant part of the PEMFC stack by means of volume, weight, and costs, the pursuit of the most suitable and least expensive metal and alloy materials, such as copper, nickel, titanium, and aluminum alloys, are proposed, emphasizing the most important family of material candidates—stainless steels [317]. Support materials are also of great interest in order to improve the activity and stability of the catalysts [318]. Low and high surface areas of semiconducting TiO_2_ and Al_2_O_3_ metal oxide–carbon hybrid catalyst support has been evaluated in single PEM fuel cells [319]. High and low surface areas of mesoporous TiO_2_ (250 m^2^/g and 45 m^2^/g, respectively) and Al_2_O_3_ (220 m^2^/g and 30 m^2^/g) were investigated as an alternate cathode catalyst support material for PEMFCs. Results revealed that a Pt catalyst supported by a high surface area TiO_2_/C (25:75) hybrid gave the most outstanding performance. Ultrathin cathode catalyst layers prepared using the decal transfer method containing carbon-supported electrocatalysts Pt/C, PtCo/C, and PtCoMn/C [320] were evaluated in terms of charge transfer resistance Rct in a single cell. The achieved high performance with MEA with ultralow Pt loading of 0.147 mg/cm^2^ was 1.42 W/cm^2^, as shown in Figure 24. The corresponding amount of platinum was 0.1035 g_Pt_/kW, which reached the index pointed out by the Department of Energy (DOE). This is considered as a promising MEA fabrication and performance for commercial applications.

### 5.1. PEMFC Performance Using Pt-Based Catalysts

**Figure 25:**
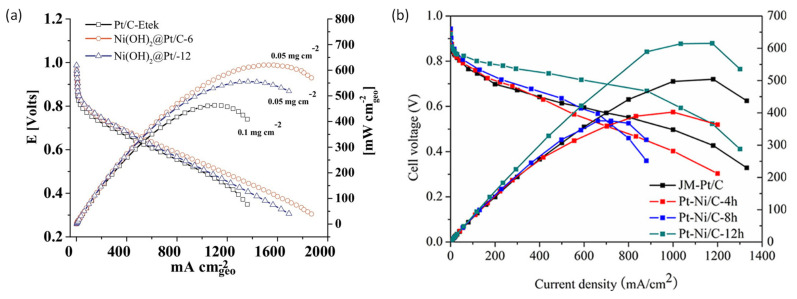
(**a**) Performance curves from single-cell configurations for Pt/C and Ni(OH)_2_@Pt/C with different catalyst loading. Reproduced with permission from Elsevier (reference [321]) and (**b**) Single-cell performance curves of different Pt-Ni cathode catalysts prepared at 200 °C for different times. Reprinted with permission from reference [296]. Copyright 2016 American Chemical Society.

The attraction for fuel cells is in their versatility, because they can be implemented across a wide range of applications from microelectronics to large-scale power generation. Cathode electrodes containing nanoparticles of Ni(OH)_2_ surrounded with ultra-low Pt content and supported on functionalized carbon, Ni(OH)_2_@Pt/C catalysts, have been reported with high activity and stability from RDE and corroborated by single-cell membrane–electrode assembly (MEA) tests, showing higher power densities with lower Pt loadings, in comparison with commercial Pt/C [321]. These results show that electrocatalysts with higher activity and stability can be obtained through precise control of the atomic-level catalyst structure, as presented in Figure 25.

Recent progress on the design and fabrication of PEMFC, with a special focus on their air-breathing planar configuration, as this extends the possibility of PEMFC to thin and flexible designs, has been reviewed [322]. Fe-N-C presented a strong performance [323], however, when tested as a catalyst-based membrane–electrolyte assembly, a degradation mechanism was presented and the cell, which performed with an initial peak power density as high as 1.1 Wcm^−2^, suffered a current loss of 52% at 0.4 V over 20 h. The experimental and DFT calculation results indicate that Fe at active sites of catalysts was attacked by hydroxyl free radicals to from H_2_O_2_, which further leached out, causing an increase in activity loss. The ionomer of the catalyst layer and the membrane was further contaminated by the leached Fe ions, which resulted in enlarged membrane resistance and cathode catalyst layer proton conduction resistance, affecting the cell performance. However, graphitic carbon nitrides (g-C_3_N_4_) [324] and hybrid Pt-e-N-C electrocatalysts [325] with unprecedented durability for fuel cells have been designed with abundant Pt and Fe single atoms homogeneously dispersed on the nitrogen-doped carbon-supported Pt-Fe alloy nanoparticles. A fuel cell with Pt-Fe-N-C as the cathode shows a larger peak power density (0.75 Wcm^−2^) than that with Fe-N-C as the cathode (0.50 Wcm^−2^). The remarkable stability and long-term durability of the cathode catalyst is reflected by no noticeable drop in the half-wave potential after 70,000 potential cycles and 80% current retention after 85 h of potential hold at 0.4 V in the fuel cell. This work demonstrated the feasibility of improving the durability of Fe-N-C material via ultra-low Pt doping, which makes non-precious metal electrocatalysts close to achieving massive production for commercial metrics. Innovative fabrication methods for the various components of planar PEMFC, as well as effective stack design and assembly, are also critical for efficiency maximization, reproducibility, and overall cost reduction. Decreasing Pt loading in the anode layer below ∼0.025 mg·cm^−2^ has been found to reduce the hydrogen oxidation reaction rate in PEMFCs under normal operation conditions when using conventional Pt/C catalysts and electrode coating methods. To achieve extremely low Pt loading in the anode catalyst layer while maintaining high PEMFC performance and durability, a series of MEAs with low Pt loading in the anode layer were successfully prepared and characterized using an atomic layer deposition (ALD) technique [326]. Results achieved with ultralow Pt loading, uniform Pt distribution, high MEA performance, and durability indicate that the ALD technique has great potential in developing high-performing electrocatalysts for PEMFC. Tuning the catalytic activity of core–shell structured Ir@Pt/C nanoparticles by controlling Ir core size synthesized by pulse electrochemical synthesis can enhance the cathode performance in a single PEM fuel cell application [327]. That work demonstrated that the synthesis of core–shell structured cathode catalyst layers with high performance is an alternative to fabricating MEAs with cathode ultra-low Pt loading. Multi-walled carbon nanotubes decorated by platinum catalyst Pt /MWCNT for high-temperature PEM fuel cell performance was reported and the Pt/MWCNT catalysts presented higher power density (0.360 W/cm^2^) than Pt/C (0.310 W/cm^2^) at 160 °C [328]. The results obtained show that the synthesized catalysts are suitable for moderate- and high-temperature applications. The influence of carbon support on the catalytic layer have been studied and results reveal that CNT-supported Pt catalysts favor a power density greater than in the carbon black support due to a more suitable porous structure of the catalyst layer [329].

### 5.2. PEMFC Performance Using Non-PGM Catalysts

The fuel cell electric vehicle has received great attention in the vehicular transport industry due to its high efficiency of about 60% in energy conversion and 90% pollution-free capability [330,331]. The high costs, limited durability, and sluggish inefficient oxygen reduction reaction have been identified as key challenges to overcome for the widespread commercial success of fuel cell transportation vehicles. The oxygen reaction is constrained by the kinetics of cathode catalysts and must be improved by suitable nanoengineering of low-cost electrocatalysts [332]. The specifications for a fuel cell system consist of establishing the performance, dimension, weight and size, emissions, output power, rapid start-up, and rapid response to changes in load, lifetime, and operability in intense environments and noise, which play an important role in certain applications. Great attention has been paid to replacing platinum group metals with carbon-based non-precious metal, nanoporous metals [333,334,335], heteroatom-doped carbons [336], covalent organic frameworks [337,338], non-precious metal, or precious-metal-free catalysts of inexpensive metals [339,340,341] as a promising new generation of electrocatalysts has emerged. This is due to their reduced cost, enhanced performance, and high stability and activity of fuel cell applications. Recent advances in noble-metal-free transition metal/nitrogen-doped carbon (M-NxC), including non-pyrolyzed and pyrolyzed transition metal macrocyclic compounds and recently developed Fe-NxC and Co-NxC catalysts have gained special attention. The M-NxC, cobalt/zinc dual sites coordinated with nitrogen in nanofiber catalysts and Fe/N/C catalysts have been proven to be one of the most promising substitutes for precious metal catalysts, due to their low costs and high catalytic performance [342,343,344,345,346]. The nanoporous metals employ electrodes composed of a continuous solid and pore phase, which ensures the flow of ions and electrons to the interface. In their structure, the size of the active material determines the specific area, the catalytic activity, and the distance over which a solid-state diffusion process operates, where the size of pores and porosity affect mass transport and ion conduction in the electrolyte. New perspectives on fuel cell technology were analyzed in a recent work to reduce the costs of the membrane and improve cell efficiency, durability and reliability, allowing them to compete with the traditional combustion engine [347]. Fundamentals of theory and practical operation of a PEMFC involving various mathematical models, quantum mechanics, and DFT studies were performed to support the experimental results [217,348,349]. Computational validation with semi-empirical Grimme DFT-D2 correction has been performed to support the experimental findings for graphene oxide (GO)-supported palladium (Pd)-iron (Fe) nanohybrids as a new generation electrocatalyst for proton exchange membrane fuel cells [349]. Electrochemical reported results show that GO-Pd-Fe nanohybrid catalysts (Pd: Fe = 2:1) demonstrate excellent catalytic activity as well as a higher electrochemical surface area of (58.08 m^2^/ (g Pd-Fe)^−1^, which is higher than the commercially available Pt/C catalyst with an electrochemical surface area of 37.87 m^2^/(g Pt)^−1^.

## 6. PEMFC Stack Configuration and Characterization

Developing clean and sustainable energies as alternatives to fossil fuels continues to be a strong demand within modern society. The operational parameters of a hydrogen/air micro PEMFC with different flow configurations (mesh, serpentine, and interdigitated) are electrochemically characterized, where impedance spectroscopy is widely accepted [350]. PEMFCs exhibit a wide power range, low operating temperature, high energy density, and durability for a long lifetime. These advantages, as mentioned previously, favor PEMFC for diverse applications, such as vehicle power sources, portable power, residential and backup power applications. Nevertheless, improper operating conditions can severely affect the fuel cell lifespan [351]. With the push towards the commercialization of PEMFC, especially for portable power applications, the overall balance of plants (BOPs) of the systems should be minimized. To reduce the mass and complexity of the systems, air breathing PEMFC stack design with open cathode channel configuration is being developed. The open cathode channel configuration incurs hydrogen leakage problems. Water and thermal management are critical for the performance and operation stability of PEMFC stacks and are highly associated with the stack configurations and cathode operating parameters, which need to be optimized. In this direction, a numerical studies have been conducted with an orthogonal analytical method to investigate the effect of stack configurations and cathode operating parameters on stack performance, including power density, system efficiency, and stack uniformity [352,353]. Since major electrochemical reactions of PEMFCs for FCEV applications are closely related with the structural features of core components on the nano- and microscales, it is essential to understand key features of PEMFCs through advanced imaging techniques [354,355]. Air breathing is known to reduce the weight, volume, and the cost of PEMFCs. The thermal management of the high-powered air breathing PEMFC stacks by applying different cathode flow channel configurations was carried out to improve the stack performance and a numerical simulation was performed to verify the thermal management results. The research results showed that a combination of the 50% and 58.3% opening ratios in the air-breathing stack reduced the stack temperature and enhanced the temperature distribution uniformity, leading to a better and more stable stack performance. It was found that the stack performance was significantly improved under the assisted-air-breathing condition. By numerically modeling a simplified partial single cell, a particular heating configuration was studied for temperature distribution, emphasizing the membrane. The effects of the anode inlet temperature (AIT) and cathode inlet temperature (CIT) on the temperature distribution showed that low temperatures can be experienced near the gas inlets, especially close to the vertical edges, which are affected by the AIT and especially by the cathode inlet temperature. Thus, it is critical to control the CIT to ensure that the membrane temperature close to the cathode inlet does not reduce to a value that is detrimental to the fuel cell performance. A dynamic model of PEMFC stack has been developed by combining an electrochemical sub-model and a thermodynamic sub-model [356]. With necessary validation, it demonstrates that modeling results and experimental data are in very good agreement in terms the polarization curve and power output. By applying the dynamic model to analyze performance outputs of PEMFC stacks and applying the model to an FC hybrid vehicle powertrain configuration, it demonstrates improved PEMFC quality. Increasing the maximum current density could increase the peak power output and increase the working efficiency, although the increase of peak power is not linear in relation to the increase of maximum direct current and higher working temperature of PEMFC, which would benefit the increase of both peak power output and efficiency. Compared to working temperature, ambient temperature′s increase could also make possible an influence on power output and efficiency, though the influence would be weak. Coupling the dynamic model with a powertrain model of an FC–electric hybrid vehicle, the analysis suggests that both PEMFC stack and battery stack should have similar size for general driving conditions.

## 7. PEMFC Water Management

Reactant distribution, and heat and water management are critically important to the performance of PEMFCs, especially at the system level, where the proton transport from the anode to the cathode side is crucial [357,358,359,360,361,362,363]. Dynamic contact angle (DCA) is of fundamental importance in the numerical investigation to conduct material simulation of water management problems in PEMFCs [364,365,366,367]. The volume of fluid (VOF) method with the dynamic contact angle (DCA) was applied to simulate droplet behaviors on inclined surfaces with different droplet impact velocities, impact angles, and viscosities [368]. It was found that the droplet spreading and deformation results from the simulations were in excellent agreement with those captured in the experiments. The results also indicate that higher impact velocity and impact angle can facilitate the spreading trend at the droplet trailing edge and have no notable effects on the leading edge. The application of the movement of a water droplet in a single serpentine flow channel of PEMFC [369] with different U-turn designs [370] and the online adaptive diagnostic strategy were reported [371] as a data-driven strategy for characterizing the water management failure in fuel cells. The original single-cell voltages were projected into lower-dimension features by applying orthogonal linear discriminant analysis, and the efficiency and reliability of this online adaptive diagnostic strategy was validated using an exptl. database from a 90-cell PEMFC stack. A 3D numerical and experimental investigation of the baffle plate was reported as an effective way to improve reactant transport and water removal in the porous electrode of PEMFCs [372,373]. This study illustrates the importance of the water management downstream of the fuel cell. Fuel recirculation is extremely important to fuel cell performance and durability, but it seems to have not received enough consideration so far [374]. In this article, authors reviewed numerical modeling with experimental results exploring the basics and fundamentals related to flows, device design, and fuel cell performance. In PEMFCs, to favor the diffusivity and, therefore, the proton conductivity, the membrane needs to be humidified. As the performance has improved, the amount water and heat generated by reaction has increased, so water and heat management of the fuel cells is becoming more important, and the effect of reactive gas recirculation flow rate, purge interval, and duration on the performance is attained. To study the impact of water management on the performance of PEMFC commercial stacks and to obtain useful indicators for fault detection, fresh and on-field aged commercial stacks were characterized by impedance spectroscopy under various humidities and current densities [375]. Results identified four capacitive and one inductive loop attributed to charge transfer kinetics at both anode and cathode and to cathodic and anodic mass transfer limitations. The inductive loop was attributed to oscillations in the ionomer water content induced by oscillations of the amount of produced water during the measurements. The effect of the catalyst layer and cathode microporous perforation on the water management and performance was studied and reported [376]. Results revealed that under humid conditions, the perforated layers enhanced the liquid water transport under the channel regions. At high current densities, the performance was improved for the cell with perforated layers owing to the mass transport losses. The hydrophobicity of the cathode catalyst layer (CCL) affects the performance of the PEMFC by adjusting the water management of the catalyst layer, which has been studied by computational fluid dynamics simulations, where a fin-shaped flow channel with hydrophobic walls to enhance liquid water removal from the surface of gas diffusion layer has been proposed [377]. The conventional serpentine flow-field designs with the two-dimensional channel and rib configuration often cause water accumulation, thus blocking the transport of reactants and interfering with the removal of water, which in turn results in reduced fuel cell performance at high current densities. Hydrophilic polymer grafting into the patterned region of three-dimensional multilayered graphene (MLG)-coated Ni foam was proposed to improve water management in fuel cells [378], along with a honeycomb flow field pattern at the cathode side [379] composed of a regular pattern of hexagonal pins that are categorized into pin-type flow fields. Results indicated that water content was less than 14% in the membrane, reducing the possibility of water flooding in the catalyst layer. To minimize water mitigation of cathodic flooding, the adoption of novel porous inserts in the flow channel of PEMFC was adopted [380]. Using a porous sponge insert (PSI) increased the power density by 23.33% for a 2 mm porous insert and the power density increased by 21.73% for a 4 mm PSI in a modified serpentine flow field. Increasing the size of the PSI from 2 to 4 mm increased the power density by 26.12% in modified serpentine flow field. The effect of the hydrophobicity of Fe-N-C CCL on the performance of the PEMFC was investigated and the authors concluded that the hydrophobicity of the Fe-N-C CLL has a great influence on the power density of the fuel cell, but the influence on the long-term durability of the fuel cell is negligible [381].

## 8. Hydrophobicity of Electrodes and Gas Diffusion Layers

**Figure 26:**
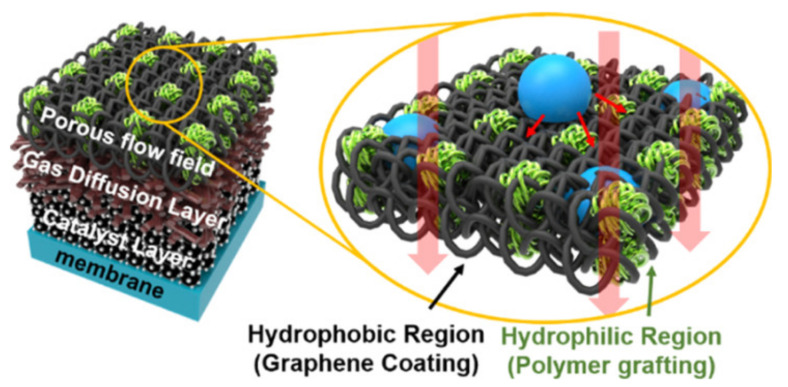
Schematic selective grafting of a hydrophilic polymer on MLG-coated Ni foam. Reprinted with permission from reference [378]. Copyright 2016 American Chemical Society.

Hydrophobicity and porosity of the gas diffusion layer (GDL) are key parameters in optimizing the design of PEMFCs. Water management is one of the obstacles in the development and commercialization of this kind of large-scale power generation. The conventional serpentine flow-field designs with the two-dimensional channel and rib configuration often cause water accumulation, thus blocking the transport of reactants and interfering with the removal of water, which in turn results in reduced fuel cell performance at high current densities. Sufficient humidification of the membrane directly affects the PEMFC performance. The balance between preventing water flooding, effective water removal, adequate humidification of the membrane, and efficient transport in the flow-field channel will provide a significant contribution to fuel cell performance [379,382,383,384]. Hydrophilic polymer insertion into the patterned region of three-dimensional multilayered graphene (MLG)-coated Ni foam was proposed to improve water management in fuel cells (Figure 26). The introduction, for the first time, of hydrophobic spherical polytetrafluoroethylene (PTFE) particles into the CNT-based catalyst layer improved the diffusion of oxygen and water and was effective during low-humidity operation of a PEMFC [385].

Modification of metallic bipolar plates with vertical carbon nanotube films grown on stainless steel improves the hydrophobic and anti-icing properties [386] of ZrC-coating-modified Ti plates [387]. Flow channels with hydrophobic walls enhance liquid water removal [377]. A hydrophobic fin-shaped flow channel can remove water droplets on the GDL and accelerate their transport, avoiding water accumulation. The interaction of microporous layers consisting of different ratios of acetylene black and carbon fibers with either a hydrophobic polytetrafluoroethylene (PTFE) or a hydrophilic perfluorosulfonic acid (PFSA) ionomer binder was studied to improve oxygen and water transport through microporous layers [388]. The effect of the nature of the substrate, carbon black, and PTFE loading in the different carbonaceous phases in the microporous layer (MPL) is usually chosen to modify their surfaces with hydrophobic materials for better H_2_O removal ability [389,390]. The effect of the hydrophobicity of microporous layers with different decorative patterns to optimize the interface of the catalyst layer and gas diffusion layer (GDL) in the performance of PEMFC was investigated [391]. Results concluded that the microporous layer could be considered as a promising strategy to improve the mass transfer efficiency at the interface of the catalyst layer and gas diffusion layer. The effect of the gas diffusion layer’s surface wettability in a serpentine gas flow has been simulated [392]. It was observed that water coverage is always lesser for a gradual hydrophobic surface. Also, at low air velocity and gradual hydrophobic GDL, the surface results in a lesser pressure drop as well as lower water coverage. Numerical investigations have been reported where two important parameters—water coverage ratio and pressure drop—have been studied in detail. The 3D unsteady-state models are used to study the drop dynamics using commercial CFD software ANSYS FLUENT 18 [393]. Simulation studies supported by experiments have been studied, analyzing the influence of hydrophobicity and porosity of the GDL on mass transport losses in PEMFC [394,395].

## 9. Performance Inhibition of PEMFCs by Electrodes Processes

An electrode is a critical component in PEMFCs as it simultaneously controls the reactant supply, proton and electron transport, water and heat management, and electrochemical reactions. Pt is the most popular catalyst due to its excellent activity, selectivity, and stability, and is required to accelerate the electrochemical reactions during the cell operation [396,397,398,399]. Electrode structure determines the rate of transport and electrochemical reactions and is significantly affected by the catalyst deposition method. The effect of catalyst deposition was investigated on the pore structure, mass transport, and operating performance of the catalyzed electrodes prepared by the methods of catalyst coated on membrane (CCM) and catalyst coated on substrate (CCS). The results indicated that the CCS electrode is thinner, yielding larger porosity, smaller geometric pore surface area, smaller diffusion and permeation resistivity, and lower cell performance. The maximum power density of the CCS electrodes is only about 4% smaller than that of the CCM electrodes at high Pt loadings (0.4 mg·cm^−2^), while it is as much as 60% less than that of the CCM counterparts at low Pt loadings (0.1 mg·cm^−2^). The significant performance drop for the low-Pt-loading CCS electrodes is due to the relatively low surface area in the catalyst layers, resulting from catalyst penetration into the pores of the gas diffusion layer, even though the mass transfer resistivity is smaller than their CCM counterparts. The CCS method is, therefore, unsuitable for low-Pt-loading electrodes (<0.1 mg·cm^−2^) unless the material penetration and the resulting performance deterioration can be inhibited.

The high costs of PEMFCs remain a roadblock for a competitive market with combustion engine vehicles, and the PEMFC costs can be reduced by decreasing and optimizing the size of Pt nanoparticles in the catalyst layer, thereby increasing the Pt dispersion and utilization, which should be taken into account for practical consideration. The high-power performance loss due to oxygen transport resistance is alleviated by decreasing the particle size and increasing dispersion [400]. The particle size of Pt nanoparticles varied from 2.0 to 2.8 and 3.7 nm while keeping the loading constant (30 wt%) on a Vulcan support using the two-step surfactant-free toolbox method. By studying the electrochemical dissolution in situ using online inductively coupled plasma mass spectrometry (online ICP-MS), mass-specific dissolution trends were revealed and attributed to particle-size-dependent changes in the electrochemically active surface area. Such degradation trends are critical for the start/stop of PEMFCs and currently require the implementation of potential control systems in consumer vehicles. Shifts in the onset of anodic dissolution and oxidation to more negative potentials with decreasing particle size were observed. These results indicate a similar mechanism of anodic dissolution related to place-exchange when moving from extended polycrystalline Pt to nanoparticle scales.

Cracks in catalyst layers (CLs) serve as gateway for water and reactive gases leaching in fuel cells. It is known how significantly this affects the performance and durability on low- and high-temperature polymer electrolyte membrane fuel cells (HT-PEMFCs). Thus, it is an important issue to control the cracks formation in catalyst layers. A simple and effective way has been developed to minimize cracks via introducing carbon nanotubes (CNTs) into CLs [401]. The introduction of CNTs reinforces the structure of CLs with small cracks. It also regulates the pore structure of cathode CLs with reduced reactive species and product invading and enhancement of mass transfer.

## 10. PEMFC Technology, Cost, Actual Applications, and Challenges

**Figure 27:**
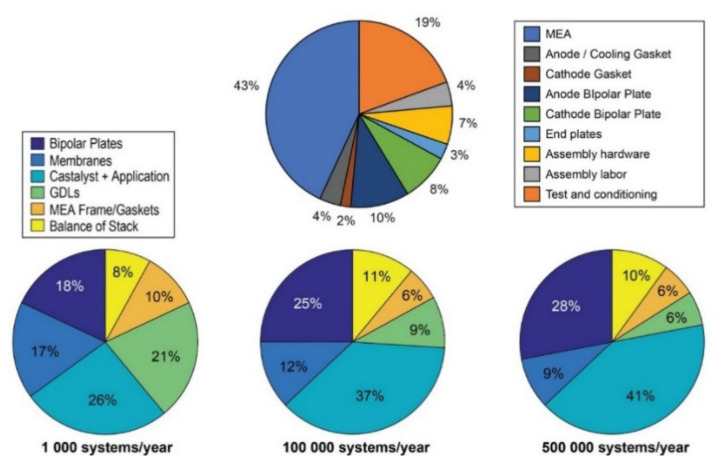
DOE cost analysis of parts, components, and systems. From references [403,404].

The energy industry for electrical energy conversion and storage has expanded globally and requires significant improvements in the PEMFC and battery technologies to address the challenges associated with cost, performance, and durability [402]. The energy conversion and storage are important links between the energy production and energy consumption [346]. As the rapid evolution of the fuel cells industry continues, it has become increasingly important to understand how varying technologies compare in terms of cost and performance. Cost undoubtedly plays a very important role in the commercialization of portable power supply and stationary fuel cell systems worldwide. The cost estimated by the DOE hydrogen and fuel cell system in 2017 [403] and the US Department of Energy [404], considering different volumes of PEMFCs manufactured per year, is shown in Figure 27. Data reported and different key assumptions of cost of catalysts, membranes, parts, and components of the fuel cell system were taken into account.

The cost of stationary fuel cell systems operating currently is normally between 25,000 and 40,000 euros per kW. Transitioning away from vehicles using conventional fossil fuel is a very promising strategy to achieve sustainable road transport. In this regard, fuel cell electric vehicles (FCEVs) are a good option because they use hydrogen fuel, which is stored on board and converted to electricity by a fuel cell [405]. The costs in energy production and storage continue to fall and opportunities in consumer, transportation, and grid applications are defined. As the rapid evolution of the fuel cell production and hydrogen storage industries continues, it has become increasingly important to understand how varying technologies compare in terms of cost, performance, and durability. Recently, experts assessed the cost and expected future performance of PEMFCs for vehicular transport [406] and concluded that they still lack wide market acceptance in vehicles. The 39 experts assessed the median 2017 automotive cost to be $75/kW, stack durability to be 4,000 hours, and stack power density to be 2.5 kW/L. However, experts ranged widely in their assessments. In 2017, the best cost assessments ranged from $40 to $500/kW, durability ranged from 1200 to 12,000 hours, and power density assessments ranged from 0.5 to 4 kW/L. Most respondents expected the 2020 cost to fall short of the 2020 target of the US Department of Energy (DOE). They identified Pt, membrane, and BPP costs as significant barriers to reducing system costs. It was described in this article [407] how several countries have aimed to increase FCEVs. Japan aims to produce 200,000 automotive FCEVs cumulative by 2025, the United Kingdom aims to produce tens of thousands of FCEVs (cars, trucks, and buses) cumulative by 2025, and China aims to produce 1 million FCEVs cumulative by 2030. Uncertainty characterizes PEMFCs’ path to widespread commercialization.

The fuel cell technology office (FCTO) established that the fuel cell market, for example, has maintained a consistent growth in the last few years, with nearly 70,000 fuel cell systems and 800 MW in fuel cell power shipped worldwide in 2018, with approximately USD $2.3 billion in fuel cell revenue. Globally, there are more than 300,000 stationary fuel cells in operation, 14,000 hydrogen-powered fuel cell cars on the road, and approximately 300 hydrogen-refueling stations [407]. In the United States, there are currently more than 500 MW of stationary fuel cells, more than 7800 fuel cell cars, and more than 28,000 hydrogen fuel cell forklifts operating at major companies. In medium-/heavy-duty transportation, the DOE′s FCTO has suggested an ultimate price target of $30/KW by 2050 for an 80 kW PEMFC system established to enable long-term competitiveness of light-duty hydrogen fuel cell cars. The state-of-the-art technology is $180/kW, a cost that could be estimated at $50/kW with low-volume production and $45/kW with high-volume projection. Reaching the ultimate target will depend on achieving economies of scale in manufacturing, coupled with continued R&D to reduce costs, including fundamental research to develop new materials for lower-cost catalysts, membranes, ionomers, and balance-of-system materials for fuel cells [408]. Further, durability and cost are both related to catalyst loading, and the challenge is to develop low-cost platinum-group-metal-free catalysts and low-PGM electrocatalysts in electrodes. One method to decrease the amount of Pt content is to increase the Pt surface area through better dispersions and smaller particle sizes or use Pt-free Fe-N-C macrocyclic compounds. Larger particles have shown better durability and particle size has been identified as one of the main properties determining durability [409].

## 11. Conclusion and Future Outlook

In summary, fuel cells are electrochemical, ecologically clean, efficient, and low-emission devices with various potential applications due to their to their high efficiency, high power density, low emissions, and energy supply. In recent years, PEMFCs have attracted increasing attention given their advantages for the automotive industry, which has been materialized in their remarkable technical progress. The technical advances provided in the last decade have created the possibility of adapting fuel cells to multiple applications, providing a great boost in their manufacture on an industrial scale. With its operation on a global scale, science hopes that humanity can generate electricity in a simple and sustainable way with the intention of reducing CO_2_ and CO pollution on the planet, which will also result in alleviating climate change, even more so in accordance with the increase that we are making in the use of electricity. All this is justified because both the storage and transport of energy can be carried out by means of hydrogen as an energy vector, since it can be stored and transported in a simple way. With the use of fuel cells and hydrogen technology, electrical energy from renewable energy sources can be supplied where and when necessary in a clean, efficient, and sustainable way, which will have an impact on making the future that our planet earth can provide more comfortable.

This review analyzed the development of high-performance polymeric membranes together with nanomaterials in terms of high catalytic activity and stability in order to reduce the platinum group metal applied as cathodes to build stacks of proton exchange membranes fuel cells (PEMFCs) to work at low and moderate temperatures. Several composite membranes have been developed in recent years for use in PEM technologies; in this vein, we found composite fluorinated, with organic and inorganic fillers, and non-fluorinated membranes. All materials reported in this review show promising characteristics and results, so it is not possible to indicate which one is the best. It can be noted that papers reporting high performance are dealing with the incorporation of fillers into the Nafion matrix, suggesting that Nafion cannot be completely replaced yet. Other materials like SPEEK and PVA are used to completely substitute Nafion. They seem to be a promising alternative to obtain high performance membranes. Beyond the use of organic fillers like ionic liquids or MOFs, other fillers, such as carbon-based materials and inorganic fillers, are the most promising materials. In this regard, the GO composite membrane extends the operating temperature range for hydrogen PEMFC since GO retains more water, so it decreases the loss in proton conductivity. Among all the composite membranes described in this review paper, inorganic fillers are the most versatile materials; their good thermal stability, improved water uptake, and reduced methanol absorbance provide a high power density. Ionic liquids can be potentially used at intermediate temperatures once performance increases. Despite these positive results, durability tests are necessary to understand the real capacity of those fillers. Research activities on their potentialities are still ongoing; particularly to resolve the difficult problem of simultaneously enhancing membrane proton conductivity and nanoparticles (NPs). The most important challenge is the design of catalytic material nanostructures and which morphologies interact efficiently with the polymeric matrix of the ionic exchange membrane. The challenges facing the scientific community are mainly found in relation to nanoparticles and which morphologies have been widely studied for catalysis applications focused on the stabilizing agents and their potential impacts in nanomaterial synthesis to induce changes in the morphology of NPs that will be efficient for the membrane surface.

The development of novel PEMFCs might help to achieve higher power densities, that is, increasing the power density from the current value of about 4 kW l^−1^ to the short-term target of 6 kW l^−1^ in 2030, and to the long-term target of 9 kW l^−1^ durability by 2040 [410]. In the next decades, PFSA-based polymers with enhanced chemical stability are expected to continue to dominate the PEM scenario, but PBI-based polymers with enhanced proton conductivity are expected to access the PEM market and settle as HT-PEMFCs.

## Figures and Tables

**Table 1:** Kinetic current for Pt and intermetallic Pt-M in 0.15 HClO_4_ [276].

Material	I_k_ (mAcm^−2^)
Pt	55
Pt-Mn	15
Pt-Pb	0.93
Pt-Sb	115
Pt-Sn	69

**Table 2:** The recent noble metal catalyst for HOR in acid medium.

Catalyst	Synthesis Method	Size Particle (nm)	i_o_ (mAcm^−2^)	Specific Activity@ 0.1 V (A/g_Ir_)	Ref.
Pd/C	Pulse microwave assisted polyol	4.3 ± 0.3	0.35	N.R.	[281]
Pd_3_Ir/C		4.5 ± 0.3	0.7	N.R.	
PdIr/C		4.4 ± 0.3	1.6	N.R.	
PdIr_3_/C		5.3 ± 0.3	1	N.R.	
Ir/C		5.7 ± 0.3	0.2	N.R.	
Pd/C	Thermal synthesis (300°C, Ar, 1h)	4.5	N.R.	N.R.	[284]
PdP_2_		5	N.R.	N.R.	
Pd_5_P_2_		5.5	N.R.	N.R.	
IrFe/C	Solvent vaporization + hydrogen reduction method	3.8 ± 0.2	N.R.	146.9	[285]
IrCo/C		2.6 ± 0.2	N.R.	133	
IrNi/C		3.4 ± 0.2	N.R.	152	
Pt		N.R.	N.R.	16	

N.R. = not reported.

**Table 3:** The recent noble metal catalyst with non-conventional supports for HOR in acid medium.

Catalyst	Synthesis Method	Size Particle (nm)	Max. Power Density (mWcm^−2^)	Ref.
Pd-Co/gCN	Thermal condensation+ polyol reduction	10	290	[282]
Pd_3_Co/PCNT	CCVD+ modified Hummers method+ polyol reduction	N.R.	327	[283]
Pd_3_Co/CNT			N.R.	
IrP_2_/rGO	Modified Hummers method+ solvothermal method	10	N.R.	[286]
Rh-Rh_2_O_3_/C	Polyol method+ thermal treatment	10-15	N.R.	[287]

N.R. = not reported.
